# m^6^A promotes planarian regeneration

**DOI:** 10.1111/cpr.13481

**Published:** 2023-04-21

**Authors:** Guanshen Cui, Jia‐Yi Zhou, Xin‐Yang Ge, Bao‐Fa Sun, Ge‐Ge Song, Xing Wang, Xiu‐Zhi Wang, Rui Zhang, Hai‐Lin Wang, Qing Jing, Magdalena J. Koziol, Yong‐Liang Zhao, An Zeng, Wei‐Qi Zhang, Da‐Li Han, Yun‐Gui Yang, Ying Yang

**Affiliations:** ^1^ CAS Key Laboratory of Genomic and Precision Medicine, Collaborative Innovation Center of Genetics and Development, College of Future Technology Beijing Institute of Genomics, Chinese Academy of Sciences and China National Center for Bioinformation Beijing China; ^2^ Sino‐Danish College University of Chinese Academy of Sciences Beijing China; ^3^ Institute of Stem Cell and Regeneration Chinese Academy of Sciences Beijing China; ^4^ State Key Laboratory of Environmental Chemistry and Ecotoxicology Research Center for Eco‐Environmental Sciences, Chinese Academy of Sciences Beijing China; ^5^ Shanghai Jiao Tong University School of Medicine & CAS Key Laboratory of Tissue Microenvironment and Tumor, Shanghai Institute of Nutrition and Health, Chinese Academy of Sciences Shanghai China; ^6^ Chinese Institute for Brain Research (Beijing), Research Unit of Medical Neurobiology Chinese Academy of Medical Sciences Beijing China; ^7^ The State Key Laboratory of Cell Biology, CAS Center for Excellence in Molecular Cell Science, Shanghai Institute of Biochemistry and Cell Biology, Chinese Academy of Sciences University of Chinese Academy of Sciences Shanghai China

## Abstract

Regeneration is the regrowth of damaged tissues or organs, a vital process in response to damages from primitive organisms to higher mammals. Planarian possesses active whole‐body regenerative capability owing to its vast reservoir of adult stem cells, neoblasts, providing an ideal model to delineate the underlying mechanisms for regeneration. RNA *N* ^6^‐methyladenosine (m^6^A) modification participates in many biological processes, including stem cell self‐renewal and differentiation, in particular the regeneration of haematopoietic stem cells and axons. However, how m^6^A controls regeneration at the whole‐organism level remains largely unknown. Here, we demonstrate that the depletion of m^6^A methyltransferase regulatory subunit *wtap* abolishes planarian regeneration, potentially through regulating genes related to cell–cell communication and cell cycle. Single‐cell RNA‐seq (scRNA‐seq) analysis unveils that the *wtap* knockdown induces a unique type of neural progenitor‐like cells (NP‐like cells), characterized by specific expression of the cell–cell communication ligand *grn*. Intriguingly, the depletion of m^6^A‐modified transcripts *grn*, *cdk9* or *cdk7* partially rescues the defective regeneration of planarian caused by *wtap* knockdown. Overall, our study reveals an indispensable role of m^6^A modification in regulating whole‐organism regeneration.

## Graphical abstract

As depicted in our model, during planarian homeostasis, the expression level of *grn* is held at a moderate level to inhibit overgrowth, as equivalent to *grn* expression level before amputation at 0 hpa. Upon injury, m^6^A modification selectively targets several important gene transcripts, including *grn* for degradation, manifested as its reduced expression level after 6 hpa, as well as transcripts of cell cycle‐related genes including *ccnt1*, *cdk7* and *cdk9*. m^6^A‐mediated downregulation of these genes, especially the *grn*, may halt the initial proliferation of the stem cell pool at the first stage of regeneration, which in turn promotes their differentiation process, including the differentiation of neoblasts to the progenitor cells and eventually to different tissue types. After *wtap* knockdown, *grn* transcript accumulates to form a unique cell cluster resulting in increased GRN secretion, which may lead to cell cycle dysregulation and the loss of neoblast differentiation and the failure of planarian regeneration.

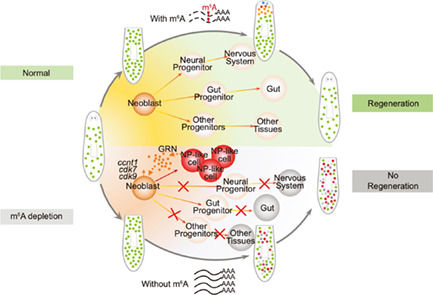

## 1 INTRODUCTION

Regeneration is a process of replacing or restoring damaged or missing cells and tissues to full function, which widely exists in the animal kingdoms.^1^ For example, hydra can regenerate from tiny body fragments to entire organisms,^1^ and zebrafish can regenerate large portions of the heart.^2^ For humans, some tissues also have regenerative potential throughout life, including the liver and muscle.^3^

Planarians are being considered a popular model system to study regeneration at the whole‐organism level.^4^, ^5^ The discovery of the planarian stem cell (neoblast) marker *piwi (smedwi‐1)*,^6^ the development of neoblast isolation^7^ and RNA interference (RNAi) knockdown^8^ methods have opened up opportunities to unravel the mechanisms of planarian regeneration. A number of pathways and regulatory factors have been identified to be essential for planarian regeneration, such as Wnt^9^, ^10^, ^11^, ^12^, ^13^, ^14^ and EGFR signalling.^15^ Especially along with the advances of third‐generation DNA^16^ and large‐scale single‐cell RNA sequencing technologies,^17^, ^18^ accumulating evidence suggests that the pluripotent neoblasts are cellular sources for regeneration.^19^, ^20^ In addition, some studies have revealed that epigenetic modifications also exert functions in planarian regeneration. For instance, the COMPASS family MLL3/4 histone methyltransferases are essential for the differentiation and regeneration of the planarian.^21^, ^22^, ^23^ In addition, the CREB‐binding protein (CBP) and p300 family of histone acetyltransferases homologues *Smed*‐CBP2 and *Smed*‐CBP3 displayed distinct roles in stem cell maintenance and functions.^24^, ^25^

*N* ^6^‐methyladenosine (m^6^A), the most abundant dynamic internal mRNA modification, has been shown to be an epi‐transcriptomic marker playing diverse regulatory roles under physiological and/or pathological conditions.^26^ Studies in various model systems have revealed that m^6^A regulates stem cell self‐renewal and differentiation.^27^, ^28^, ^29^, ^30^ Depletion of either m^6^A methyltransferases or its demethylases dramatically affects gene expression profiles.^31^, ^32^, ^33^, ^34^ High‐throughput sequencing technologies enable the detection of the m^6^A location within the transcriptome.^31^, ^35^ Subsequently, m^6^A has been identified in various RNA species and has been implicated in stem cell biology, developmental and cancer biology.^26^ For example, two separate studies showed that m^6^A regulates the transition of embryonic stem cells from a pluripotent state to a differentiated state. In this case, m^6^A selectively marks transcripts that code for key transcription factors involved in differentiation.^27^, ^36^ This demonstrates that m^6^A is a molecular switch that regulates stem cell differentiation, a fundamental mechanism in development and stem cell biology. Moreover, recent work showed that m^6^A regulates haematopoietic stem cell regeneration^37^ and axon regeneration in the mouse nervous system.^38^ While those previous studies have provided important insights into the underlying molecular mechanism of m^6^A‐mediated regulation of regeneration at the cellular level, whether and how m^6^A is implicated in regeneration in tissues and entire organisms is unknown.

Here, we employed planarian *Schmidtea mediterranea* as the model to investigate the role of m^6^A in stem cell function and whole‐body regeneration. The results illustrated that depletion of the m^6^A methyltransferase regulatory subunit *wtap* abolished planarian regeneration, which is mainly mediated by *wtap*‐dependent m^6^A genes functioning in cell–cell communication and cell cycle through m^6^A‐seq analysis. Further, single‐cell RNA‐seq (scRNA‐seq) demonstrated that a unique type of neural progenitor‐like cells (NP‐like cells) specifically expressing a cell–cell communication ligand granulin (*grn*) was induced upon *wtap* depletion. Intriguingly, depletion of *wtap* with any one of GRN, cyclin‐dependent kinase 9 (CDK9) and CDK7, a signalling axis modified by m^6^A, partially rescues the defective regeneration of *wtap* knockdown planarian. Collectively, we reveal an indispensable role of m^6^A for whole‐organism regeneration. These findings improve our current understanding of the critical molecular events controlling regeneration and have the potential to benefit future cell‐ or tissue‐based replacement therapies.

## 2 RESULTS

### 2.1 m^6^A methyltransferase complex upregulated during planarian regeneration

**FIGURE 1:**
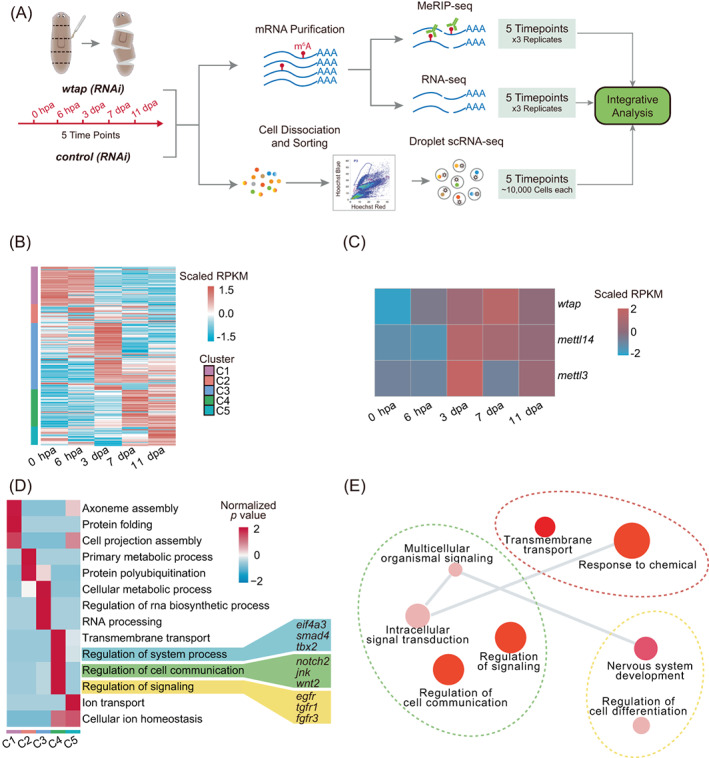
m^6^A methyltransferase complex upregulated during planarian regeneration. (A) Experimental outline of multi‐omics data including methylated RNA immunoprecipitation sequencing (MeRIP‐seq), bulk RNA‐seq and single‐cell RNA‐seq. (B) Heatmap showing the expression levels of all expressed genes and clustered into five categories based on expression pattern during planarian regeneration (left side of the heatmap). Genes were clustered by MEV with parameter—distance‐metric‐selection = Pearson correlation, number of cluster = 5, maximum iterations = 50 and plot with the parameter ‘pheatmap (matrix, scale =“row”)’ through pheatmap function (R package). (C) Heatmap showing the expression levels of major components of m^6^A writers during regeneration. (D) Heatmap showing the enriched gene ontology (GO) and important genes of five different categories as shown in (B). The colour bar of heatmap represents *z*‐score of GO's *p*‐value. (E) Gene ontology of genes belongs to fourth category (C4) shown in (B). Visualization was performed using Revigo with default parameters (semantic similarity measure = SimRel).

During planarian regeneration, gene expression profiles of all regeneration time points undergo continuous changes that can be represented by three distinct timepoints including 6 h post‐amputation (hpa), 3 days post‐amputation (dpa), and 7 dpa.^20^ These timepoints display a unique composition of *piwi‐1* (*smedwi‐1*) positive population and *smedwi‐1* negative population,^20^ resembling the combination of both processes of tissue regeneration from proliferating neoblasts (epimorphosis) and remodelling of the existing tissues (morphallaxis).^39^ In order to gain an overview of the gene expression dynamics from our own perspective during planarian regeneration, we performed RNA‐seq at five different time points after amputation (0 hpa, 6 hpa, 3 dpa, 7 dpa, 11 dpa; 0 hpa means sample collected immediately after amputation; Figure 1A). To investigate the gene expression changes of the planarian during regeneration, we grouped all the expressed genes into five clusters according to their distinct expression pattern, with each cluster containing groups of genes upregulated specifically at a certain timepoint after amputation (Figure 1B). Since m^6^A modification is catalysed by three key components of the methyltransferases, including *mettl3*, *mettl14* and indispensable regulatory component *wtap*,^40^, ^41^ we found that all these three genes displayed upregulated expression at 3, 7 and 11 dpa (Figure 1C), and all belong to the clusters 3 and 4. Based on the gene ontology (GO) functional analysis, several genes in cluster 4 were observed to be related to the regulation of system process, signalling and cell communication (Figure 1D, Table S1), including growth factors and transcription factors such as *wnt2* and *egfr* (Figure 1D). Furthermore, GO enrichment analysis revealed that genes from cluster 4 also involve in nervous system development and differentiation (Figure 1E, Table S1). Since the components of m^6^A enzymes have a similar expression trend as genes in cluster 4, these findings suggest the potentially important roles of m^6^A in regulating planarian regeneration through the aforementioned pathways.

### 2.2 m^6^A changes dynamically during planarian regeneration

**FIGURE 2:**
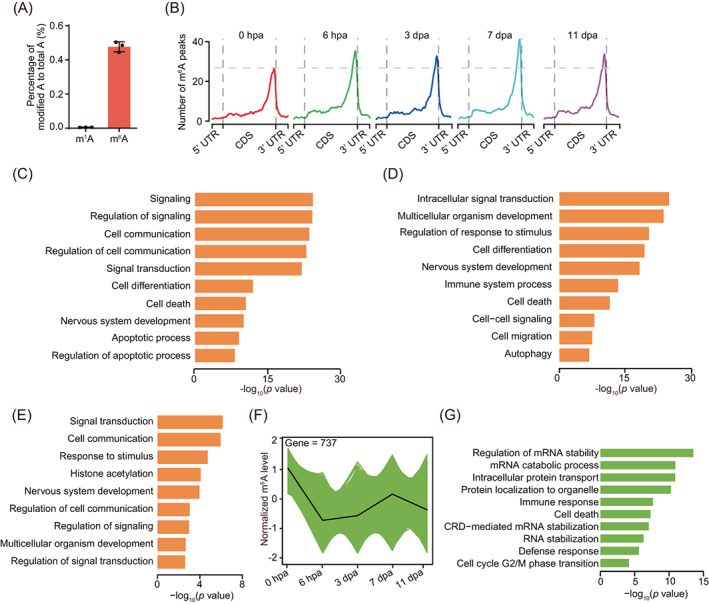
m^6^A changes dynamically during planarian regeneration. (A) Barplot showing the abundance of m^1^A and m^6^A along mRNA. The methylation level of each RNA methylation was quantified via UHPLC‐MRM‐MS/MS. The abundance of each modification was calculated with the percentage of modified A to total A. (B) Metagene profiles of m^6^A peaks along transcripts with three non‐overlapping segments (5′UTR, CDS and 3′UTR) for five regeneration stages. (C) Barplot showing the significant GO terms for 6385 mRNAs that conserved with m^6^A modification of five different timepoints shown in Figure S1C. (D) Barplot showing the significant gene ontology (GO) terms for 12,016 mRNAs that with m^6^A modification of at least one timepoint during regeneration showing in Figure S1C. (E) Barplot showing the significant GO terms for 1532 mRNAs that with m^6^A modification of four different regeneration timepoints (6 hpa, 3 dpa, 7 dpa and 11 dpa) showing in Figure S1C. (F) Line chart showing one of the trends (fourth category) of mRNAs m^6^A level during regeneration, which with gradual decreased m^6^A level from 0 hpa to 11 dpa. mRNAs with different expression pattern were defined by MEV with parameter—distance‐metric‐selection = Pearson correlation, number of cluster = 4, maximum‐iterations = 50. (G) Barplot showing the significant GO terms for mRNAs shown in Figure 2F. See also Figure S1.

To determine the regulatory role of m^6^A in planarian regeneration, we first measured the relative level of m^6^A in planarian mRNA, which shows a much higher level of over 0.5% relative to another modification on adenosine (*N* ^1^‐methyladenosine, m^1^A) by using UHPLC‐MRM‐MS/MS (ultra‐high‐performance liquid chromatography‐triple quadrupole mass spectrometry coupled with multiple‐reaction monitoring; Figure 2A).

To obtain m^6^A landscape during planarian regeneration, we performed transcriptome‐wide m^6^A mapping on samples of five time points (0 hpa, 6 hpa, 3 dpa, 7 dpa, 11 dpa) of regenerative process (Figure 1A). We carried out methylated RNA immunoprecipitation sequencing (MeRIP‐seq) to investigate the features and distribution dynamics of m^6^A in mRNA. In total, we identified 9057–13,362 m^6^A peaks over all five different regeneration time points. We found that most genes have one MeRIP peak (Figure S1A). The total number of m^6^A peaks at each stage (especially at 6 hpa and 7 dpa) of regeneration is slightly higher than that at 0 hpa (Figure S1B). Consistent with previous observations in mammals,^31^ we found that m^6^A peaks were markedly enriched near the stop codon. Also, the distribution pattern of m^6^A is similar among different regeneration time points (Figure 2B). While transcripts of 6385 genes contain m^6^A peaks at all regeneration time points including the one before amputation (0 hpa), 1532 genes show transcript being modified only after amputation (i.e., only in timepoints other than 0 hpa). We can also find m^6^A‐modified transcripts that are unique to each particular time point during regeneration (Figure S1C, Table S2).

GO enrichment analysis revealed that the 6385 shared m^6^A‐modified gene transcripts are associated with various essential biological processes, including regulation of signalling, regulation of cell communication and cell differentiation (Figure 2C, Table S1). The m^6^A‐modified genes during regeneration were related to signal transduction, cell differentiation, and nervous system development (Figure 2D, Table S1), indicating an important role of m^6^A in these regeneration‐related processes. The overlap of all m^6^A‐modified transcripts with five different clusters indicates that the cluster 4 genes with upregulation at 7 and 11 dpa show the highest overlap mostly with m^6^A‐modified gene transcripts (Figure S1D). GO analysis shows that the genes with their transcripts exclusively modified during the regeneration period are related to response to stimulus, cell communication and signal transduction pathways (Figure 2E, Table S1).

By classifying genes based on different patterns of the change of m^6^A modification level during regeneration (Figures S1E‐J and S2F–G), we found that genes with m^6^A level change following the same trend of *wtap* expression change during regeneration were related to cell communication, signal transduction and system development (Figure S1E‐J, Table S1). Conversely, genes with decreased level of m^6^A during regeneration showed involvement in the regulation of mRNA stability and cell cycle G2/M phase transition‐related pathways (Figure 2F–G, Table S1). These results may imply a potential role of m^6^A in cell–cell communication and neuron development.

### 2.3 m^6^A methyltransferase *wtap* depletion impairs regeneration in planarians

**FIGURE 3:**
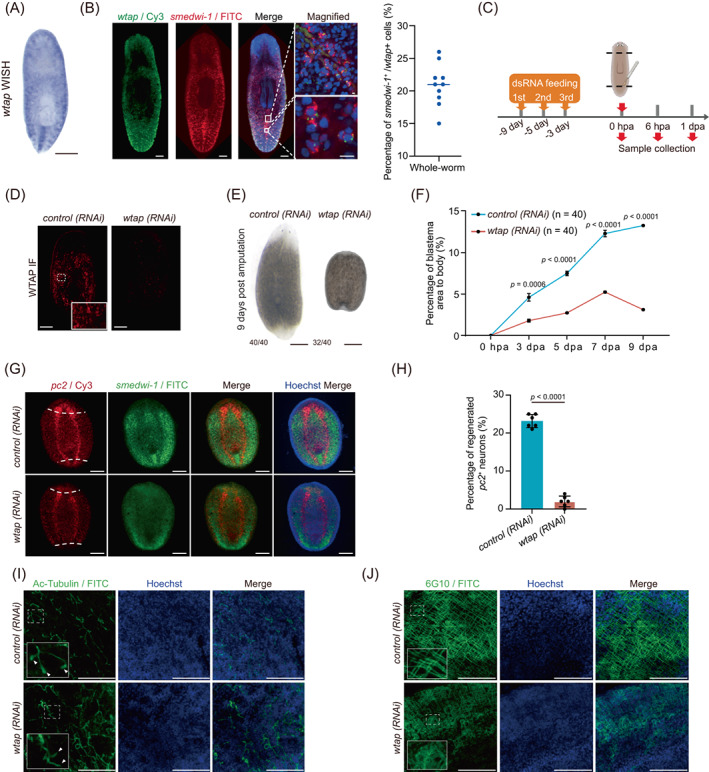
*wtap* depletion leads to regeneration defects. (A) WISH showing the expression and localization of *wtap* transcripts in planarians. Scale bar, 300 μm. (B) Whole‐mount fluorescent in situ hybridization showing the expression and localization of *wtap* and *smedwi‐1* transcripts. Scale bar, 100 μm (left panels), 10 μm (right upper row), 10 μm (right lower row). Right panel, scatter plot showing the percentage of *smedwi‐1* ^+^/*wtap* ^+^ cells. (C) Schematic diagram showing the knockdown strategy and amputation position. (D) Immunofluorescence showing expression and localization of WTAP protein in control (*control*) and *wtap* knockdown (*wtap* RNA interference [RNAi]) planarians (*n* ≥ 3). Scale bar, 200 μm. (E) Bright‐field image showing total body sizes for control (*control*) and *wtap* knockdown (*wtap* RNAi) planarians at 9 dpa. Scale bar, 500 μm. Bottom left number, planarians with phenotype of total tested. (F) Percentage of blastema area to total body size in control (*control*) and *wtap* knockdown (*wtap* RNAi) planarians. Error bars represent standard deviation. Data are the mean ± S.D (*n* ≥ 3 independent experiments). The *p* values were determined using a two‐sided unpaired Student's *t*‐test. (G) Whole‐mount fluorescent in situ hybridization showing expressions and localizations of *pc2* (red) and *smedwi‐1* (green) along whole body of control (*control*) and *wtap* knockdown (*wtap* RNAi) planarians at 7 dpa. Dotted line, amputation plane (*n* ≥ 3). Scale bar, 200 μm. (H) Quantification analysis of the percentage of regenerated *pc2* positive neuron in control (*control*) and *wtap* knockdown (*wtap* RNAi) planarians at 7 dpa. Error bars represent standard deviation. Data are the mean ± S.D. (*n* ≥ 3 independent experiments). The *p* values were determined using a two‐sided unpaired Student's *t*‐test. (I) Immunofluorescence showing the distribution of Ac‐Tubulin protein in control (*control*) and *wtap* knockdown (*wtap* RNAi) planarians at 7 dpa. Arrow indicates the presence of flame cell in the protonephridia of control (*control*) planarians, and missing in the *wtap* knockdown (*wtap* RNAi) planarians (*n* ≥ 3). Scale bar, 75 μm. (J) Immunofluorescence shows the distribution of 6G10 protein distribution in control (*control*) and *wtap* knockdown (*wtap* RNAi) planarians at 7 dpa. (n ≥ 3) Scale bar, 75 μm. See also Figure S2.

To further study the functional roles of m^6^A modification in planarians, we first performed whole‐mount in situ hybridization of m^6^A methyltransferase regulatory subunit, *wtap*, and found that *wtap* specifically colocalizes with the neoblast marker *smedwi‐1*, especially at the posterior region, suggesting a regulatory role of m^6^A in neoblast (Figure 3A,B). To study whether m^6^A modification is indispensable for planarian regeneration, the key regulatory unit, *wtap*, was knocked down by RNAi, and then the worm was amputated at both anterior and posterior regions (Figure 3C). Protein expression and mRNA levels of *wtap* were significantly reduced compared to the control, as shown by whole‐mount immunostaining (Figure 3D), western blot (Figure S2A) and quantitative reverse‐transcription PCR (qRT‐PCR) (Figure S2B). Accordingly, the mRNA m^6^A level was reduced by nearly 80% at 7 dpa (Figure S2C).

Most importantly, *wtap*‐deficient planarians failed to fully regenerate the amputated tissue parts compared to the controls, which regrow head and tail tissues at 9 dpa (Figure 3E). Moreover, newly regenerated tissue in the *wtap*‐deficient planarian area was significantly smaller at 3 dpa than that of the control planarians (Figure S2D). The blastema formed at the wound region of *wtap*‐deficient planarians was also smaller and failed to grow the missing anterior and posterior tissues in comparison with the control planarians (Figure 3F). Especially at 7 dpa, when control planarians had regrown photoreceptors, the *wtap* knockdown planarians appeared to be still unable to give rise to any new tissues from their healed wounds (Figure S2D). We noticed that *smedwi‐1* RNA expression did not change obviously in *wtap* knockdown planarians (Figure 3G). Since *smedwi‐1* RNA is one of the canonical markers of stem cells in planarians, *wtap*‐mediated m^6^A depletion may affect neoblast function. At the same time, the signatures of the level of regeneration are characterized by the regeneration of the nervous system, containing a pair of ventral nerve cords and the brain.^42^ However, neither part was regrown in *wtap* knockdown planarians, shown by whole‐mount fluorescence in situ hybridization (FISH) staining of *pc2* (Figures 3G,H and S2E), a neuropeptide processor expressed throughout the planarian central nervous system (CNS). Apart from the failure to regenerate new tissues, the existing tissues were also abnormal upon *wtap* knockdown, such as the missing ciliated ‘flame cells’ of protonephridia in the excretory system (Figure 3I) and disorganized muscle fibres (Figure 3J), which were indicated by the immunofluorescence staining of Ac‐tubulin and 6G10, respectively.

Impaired regeneration and abnormal tissue morphology may occur due to the abnormality of the proliferation of the neoblast and progenitor cells, or apoptosis of terminally differentiated cells. Thus, we investigated if proliferation and apoptosis are impaired in *wtap* knockdown planarians. We first performed BrdU pulse‐chase labelling assay and revealed BrdU‐labelled cells planarians were more enriched near the amputation site in *wtap* knockdown than that in control planarians, indicating the cell proliferation near the amputation site is more pronounced under *wtap* knockdown relative to control (Figure S2F,G). Then, we performed TUNEL assay to analyse apoptosis. The results showed a remarkable increase in cells undergoing apoptosis starting from 3 dpa in *wtap*‐deficient in comparison to control planarians (Figure S2H,I). These results indicate that m^6^A is essential for planarian regeneration, and its depletion leads to abnormal cell proliferation accompanied by increased apoptosis.

### 2.4 WTAP‐mediated m^6^A controls cell cycle‐ and cell–cell communication‐related factors essential for regeneration

**FIGURE 4:**
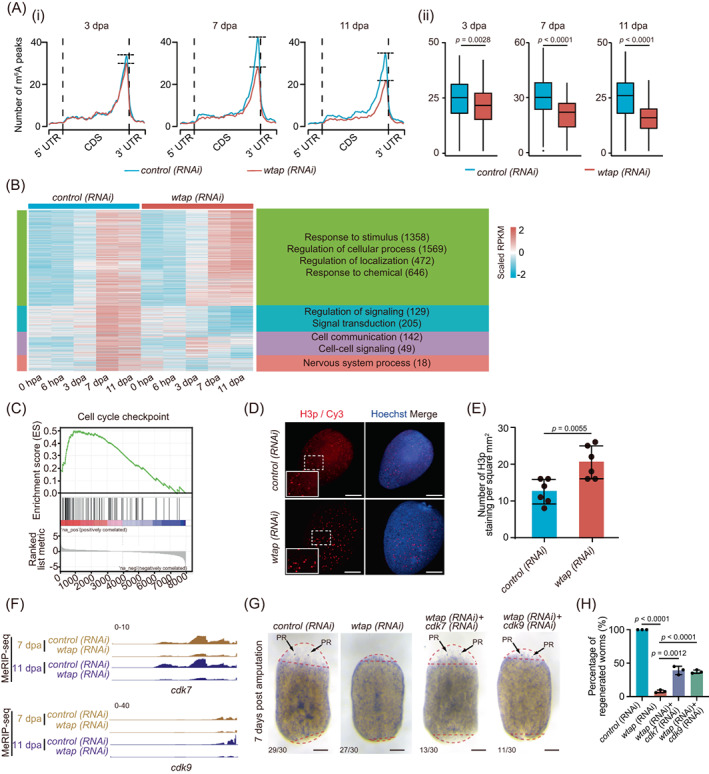
WTAP‐mediated m^6^A controls cell cycle‐ and cell–cell communication‐related factors. (A) (i) Metagene profiles of m^6^A peaks along transcripts with three non‐overlapping segments (5′‐UTR, CDS and 3′‐UTR) for *control* (blue) and *wtap* knockdown (red) planarians of different regeneration timepoints (3, 7, and 11 dpa). (ii) Boxplot showing the difference in the number of m^6^A peaks that located near stop codon of transcripts of *control* (blue) and *wtap* knockdown (red) planarians at 3, 7 and 11 dpa. The *p* values were determined using Wilcoxon‐test. (B) For genes from cluster 4 (C4) in Figure 1B, the expression level during regeneration were displayed for both *control* and *wtap* knockdown planarians of five timepoints. Then those genes were separated into four new sub‐groups based on expression features and the enriched GO terms were shown for each sub‐group. Genes with different expression pattern were defined by MEV with parameter–distance‐metric‐selection = Pearson‐correlation–number‐of‐cluster = 4–maximum‐iterations = 50. (**C**) GSEA plots evaluating the changes in cell cycle checkpoint pathway upon *wtap* depletion. Normalized *p* value <0.01. (**D**) Immunofluorescence showing the distribution of H3P protein in control (*control*) and *wtap* knockdown (*wtap* RNA interference [RNAi]) planarians at 5 dpa. Scale bar, 200 μm. Enlarged field was selected to best represent the statistical mean of staining signals. (**E**) Statistical analysis for H3p immunostaining. Error bars represent standard deviation. Data are the mean ± S.D. (*n* ≥ 3 independent experiments). The *p* values were determined using a two‐sided unpaired Student's t‐test. (F) Integrative genomics viewer tracks displaying the distributions of MeRIP‐seq data that normalized by RNA‐seq data along *cdk7 and cdk9* in both *control* (top) and *wtap* knockdown (bottom) planarians at 7 dpa and 11 dpa. (**G**) Bright‐field images showing the phenotypes of *control*, *wtap*, *cdk7* + *wtap* and *cdk9* + *wtap* knockdown planarians at 7 dpa. Scale bar, 200 μm. Bottom left number, number of planarians with phenotype versus total number tested. (H) Barplot showing percentage of regenerated worms to total sample size in control, *wtap*, *cdk7* + *wtap* and *cdk9* + *wtap* knockdown planarians at 7 dpa. Error bars represent standard deviation. Data are the mean ± S.D. (*n* ≥ 3 independent experiments). The *p* values were determined using a two‐sided unpaired Student's t‐test. See also Figure S3.

To investigate the underlying molecular mechanism for the defective planarian regeneration mediated by a decreased m^6^A level upon *wtap* knock‐down, we performed m^6^A MeRIP‐seq in control and *wtap* knockdown planarians (Figure 1A). Even though the distribution of m^6^A along the CDS only slightly differs in *wtap* knockdown compared to controls, m^6^A peaks near stop codons become visibly reduced in *wtap* knockdown compared to the control worms at each time point during regeneration (Figure 4A). We found that among the genes having upregulation trends in cluster 4 (Figure 1B), many genes showed a disordered expression pattern after *wtap* knockdown (Figure 4B). Further, GO functional analysis indicated that these genes are related to the regulation of signalling, cell communication and signal transduction‐associated pathways (Figure 4B, Table S1). We then analysed the differentially expressed genes in each time point between control and *wtap* knockdown planarians. During the regeneration process, the number of both upregulated and downregulated genes went up starting from 0 hpa with the highest number of genes changed at late stages, suggesting that *wtap* deficiency ultimately leads to regeneration failure through affecting a series of genes in multiple stages of regeneration (Figure S3A). This indicates a sustaining, persisting and specific effect of *wtap* deficiency during the regeneration process. Gene set enrichment analyses revealed a marked upregulation of the cell cycle checkpoint signature and downregulation of cell–cell signalling in *wtap* deficient samples compared to control during regeneration (Figures 4C and S3B).

We then performed immunofluorescence analysis using phosphorylated histone 3 (H3p), a typical marker for M phase, and found a significant increase in the percentage of M phase cells upon *wtap* knockdown, and no significant difference after 5 dpa in control planarians (Figure 4D, E). One of the key regulators of G2/M phase, Cyclin B1, was shown to have an elevated level of whole‐mount FISH staining during regeneration (Figure S3C). Therefore, combining the results that unaltered proliferation near the wound site but increased apoptosis upon *wtap* knockdown, we propose that *wtap* deficiency might drive the entry of the cells into the cell cycle resulting in M phase stalling and eventually cell death. We further observed that the mRNA expression of *cyclin‐dependent kinase 7* (*cdk7*) and *cdk9*, both important cell cycle‐related factors,^43^, ^44^, ^45^, ^46^, ^47^ was upregulated upon *wtap* knockdown, while the level of m^6^A modification decreased (Figure 4F). In addition, genes related to cell–cell communication, such as *notum* and *kcnd2*, were downregulated and their m^6^A level also decreased after *wtap* knockdown (Figure S3E,F). Furthermore, double knockdown of *cdk7* or *cdk9* with *wtap* partially rescues the *wtap* knockdown phenotype (Figure 4G) from 9% regenerated worms in *wtap* knockdown group up to 39% and 37% in *cdk7/wtap* and *cdk9/wtap* double knockdown groups, respectively (Figure 4H), while neither of *cdk7* nor *cdk9* single knockdown affects regeneration (Figure S3G). This indicates that *wtap* knockdown phenotype is mediated by *cdk7* or *cdk9*. Taken together, the results imply that WTAP controls planarian regeneration potentially through m^6^A‐modified genes functioning in cell–cell communication and cell cycle.

### 2.5 scRNA‐seq unveils impact of WTAP on specific cell types essential for regeneration

**FIGURE 5:**
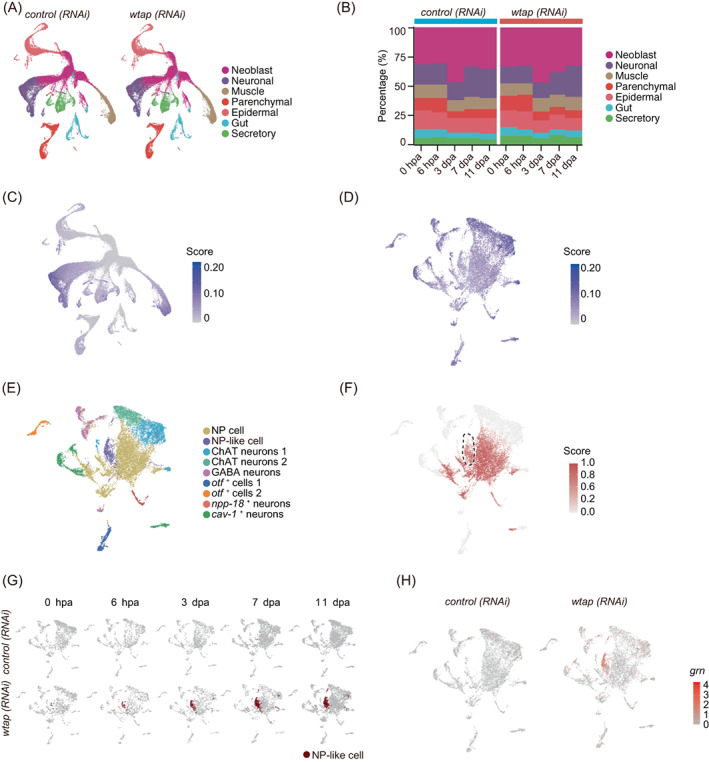
Single‐cell atlas unveils cell‐type specific regulation of WTAP essential for regeneration. (A) Based on single‐cell RNA‐seq data, Uniform Manifold Approximation and Projection (UMAP) detect major cell types in both *control* (left) and *wtap* knockdown (right) planarians of 5 timepoints during regeneration (0 hpa, 6 hpa, 3 dpa, 7 dpa and 11 dpa). Each point depicts a single cell, coloured according to cell types. (B) The percentage of each cell type in *control* (left) and *wtap* knockdown (right) planarians during regeneration. (**C**) Expression score of transcripts with m^6^A modifications are plotted onto the UMAP map. Expression score is calculated by using the *AddModuleScore* function from Seurat, and the transcripts used to score cells are from cluster 4 (C4) of Figure 1B and required with m^6^A modification. (D) Expression score of transcripts with m^6^A modifications are plotted onto the UMAP map in neuronal cells. Expression score is calculated by using the *AddModuleScore* function from Seurat, and the transcripts used to score cells are from cluster 4 (C4) of Figure 1B and required with m^6^A modification. (E) UMAP plot demonstrates nine sub‐clusters of neuronal cells in *control* and *wtap* knockdown planarians. Each point depicts a single cell, coloured according to cell types. (F) The NP cell prediction score of neuronal cells. The score is calculated by using the *TransferData* function from Seurat and the score represents the degree of similarity to neuronal progenitor cells. (**G**) UMAP plot showing a novel cell type, NP‐like cells, mainly occur in *wtap* knockdown planarians but rarely in controls. Each point depicts a single cell. (**H**) Expression level of *grn* in neuronal cells. *Control* samples on the left, *wtap* knockdown samples on the right. See also Figure S4.

To decipher the detailed mechanism of m^6^A in regulating planarian regeneration, we conducted single‐cell transcriptome sequencing of planarian *Schmidtea mediterranea* from five‐time points (0 hpa, 6 hpa, 3 dpa, 7 dpa, 11 dpa) in control and *wtap* knockdown samples. In total, 86,758 cells were successfully detected from all sequencing datasets of all samples. These cells were pooled together to generate a whole cell‐type specific atlas. To identify individual cell types, we first performed unsupervised clustering using highly variable genes, and obtained 116 clusters by the Uniform Manifold Approximation and Projection (UMAP) method. We then elucidated the cell type identity of each cluster by identifying marker genes and compared them to markers in previous studies^18^, ^20^ (Figure S4A). Overall, seven cell type groups such as neoblast, neuron, muscle, gut, secretory, epidermal and parenchymal cells were identified in both control and *wtap* knockdown samples (Figure 5A, Table S3).

In order to explore the regulation of m^6^A in cell lineage formation during planarian regeneration, we compared the cell–cell composition within control and *wtap* knockdown planarians. We found that the proportion of neural cells changes during regeneration. At 7 dpa, the proportion of regenerated neuronal cells in control planarians is about 25.03%, while it was 18.65% in *wtap* knockdown planarians, namely compared with control planarians was about 25.50% (6.38%/25.03%) decreased of neuronal cells proportion upon *wtap* knockdown. Notably, about one‐fourth of neuronal cells could not repopulate normally after *wtap* knockdown planarians (Figure 5B, Table S4). Except for neuronal cells, the distribution of the other cell types between control and *wtap* knockdown samples was similar. In order to explore the cellular functional changes after *wtap* knockdown, we compared the control and *wtap*‐deficient samples of each cell type and found that epidermal and neoblast contain the most differentially expressed genes (DEGs) in total (Table S4). GO analysis revealed that during the regeneration, after *wtap* knockdown, the upregulated genes in epidermal were enriched in the nucleic acid metabolic process and cell cycle‐related functions (Figure S4B, Table S7), and related to cell cycle regulation functions in neoblast (Figure S4C). In addition, the downregulated genes are distributed in epidermal, neoblast and neuronal cells from 3 to 11 dpa. Moreover, the downregulated genes in epidermal cells are related to the establishment of tissue polarity, translation, and ATP biosynthetic process (Figure S4D). The functions of downregulated genes in the neoblast are associated with RNA splicing, DNA replication initiation, protein folding, and so forth (Figure S4E). Neuronal cells are associated with protein folding, neurotransmitter secretion and signal release from synapse functions (Figure S4F). These data suggest that *wtap* knockdown‐induced regeneration retardation might through affecting the cell cycle and signal transduction pathways of neoblast and neuronal cells.

### 2.6 WTAP‐mediated m^6^A depletion increases GRN levels, which potentially control cell–cell communication essential for planarian regeneration

The results of single‐cell data showed that the proportion of neural system showed dominant changes after *wtap* knockdown, and the m^6^A modified genes with the same expression trend as the known m^6^A methyltransferases were enriched in the neuronal cells (Figure 5C,D). In addition, planarians could not regenerate nerve cord structure after *wtap* knockdown (Figure 3G,H). In order to explore the underlying mechanisms for abnormal regeneration of the planarian nervous system, we further classified neuronal cells according to the reported markers and also transferred the reported cluster labels onto our data by Seurat.^18^ We clustered neuronal cells into nine subtypes, neural progenitor cells (NPCs), NP‐like cells, ChAT neurons 1, ChAT neurons 2, GABA neurons, *otf* ^+^ cells 1, *otf* ^+^ cells 2, *npp‐18* ^+^ neurons, *cav‐1* ^+^ neurons (Figure 5E). Among them, we identified a unique NP‐like cell cluster during regeneration after *wtap* knockdown (Figure 5E,F), which similar to neural progenitor cells, gradually increases during regeneration but is rarely detected in the control planarians (Figure 5G). We further identified *grn* (SMED30003962), *ubiqp* (SMED30009873) and *dph1* (SMED30026267) as specific m^6^A‐modified markers of NP‐like cells (Figures 5H and S5A–D, Table S5).

**FIGURE 6:**
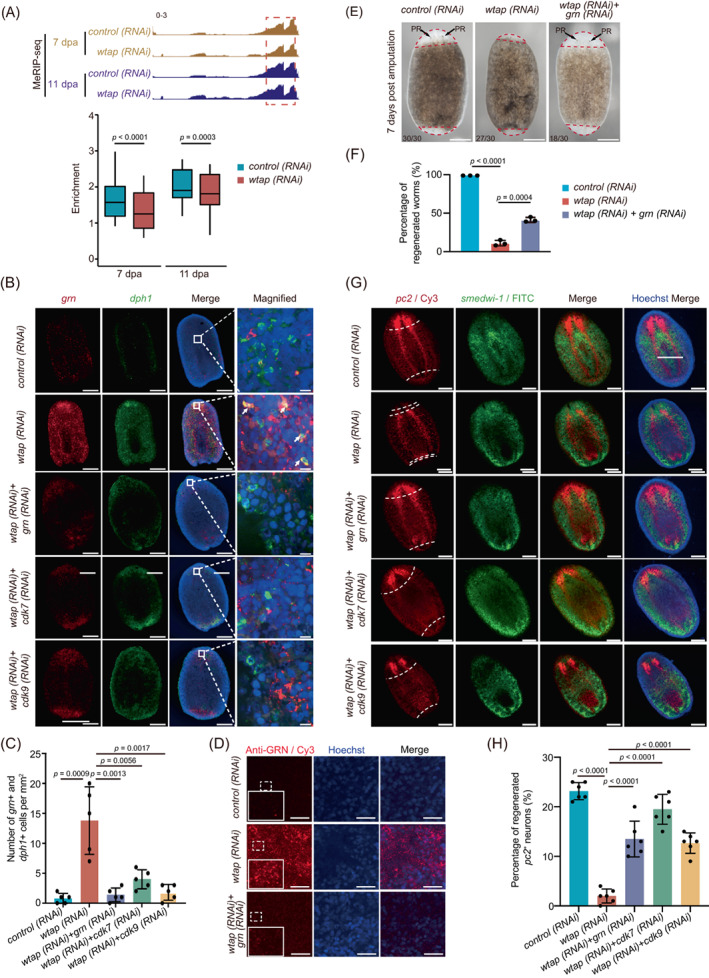
WTAP‐mediated m^6^A depletion increases GRN levels, and influences cell–cell communication essential for planarian regeneration. (A) Integrative genomics viewer tracks displaying the distributions of methylated RNA immunoprecipitation sequencing (MeRIP‐seq) data that normalized by RNA‐seq data along *grn* in both *control* and *wtap* knockdown planarians at 7 and 11 dpa (top). Barplot showing the enrichment of m^6^A modification on *grn*. The *p* values were determined using Wilcoxon‐test. (B) Whole‐mount fluorescent in situ hybridization showing expressions of *grn* (red) and *dph1* (green) in *control*, *wtap* knockdown (*wtap* RNA interference [RNAi]), double knockdown of *wtap* and *grn* (*wtap + grn* RNAi), double knockdown of *wtap* and *cdk7* (*wtap + cdk7* RNAi), double knockdown of *wtap* and *cdk9* (*wtap + cdk9* RNAi) planarians at 7 dpa. Hoechst indicates DNA staining. Scale bar, 200 μm (left panels), 10 μm (magnified panels). (C) Barplot showing the number of *grn* ^+^/*dph1* ^+^ cells of control (*control*), *wtap* knockdown (*wtap* RNAi), double knockdown of *wtap* and *grn* (*wtap + grn* RNAi), double knockdown of *wtap* and *cdk7* (*wtap + cdk7* RNAi), double knockdown of *wtap* and *cdk9* (*wtap + cdk9* RNAi) planarians in (B). Data were analysed by the two‐tailed unpaired Student's *t‐*test (bottom). (D) Immunofluorescence showing the expression and localization of GRN protein in control (*control*), *wtap* knockdown (*wtap* RNAi), double knockdown of *wtap* and *grn* (*wtap + grn* RNAi) planarians at 7 dpa. Hoechst indicates DNA staining. Scale bar, 100 μm. (E) Bright‐field images showing the phenotypes of control (*control*), *wtap* knockdown (*wtap* RNAi), double knockdown of *wtap* and *grn* (*wtap + grn* RNAi) planarians at 7 dpa. Scale bar, 300 μm. Bottom left number, number of planarians with phenotype versus total number tested. (F) Barplot showing percentage of regenerated worms in control (*control*), *wtap* knockdown (*wtap* RNAi), double knockdown of *wtap* and *grn* (*wtap + grn* RNAi) planarians at 7 dpa. Error bars represent standard deviation. Data are the mean ± SD (*n* ≥ 3 independent experiments). The *p* values were determined using a two‐sided unpaired Student's *t*‐test. (G) Whole‐mount fluorescent in situ hybridization showing expressions and localizations of *pc2* (red) and *smedwi‐1* (green) of control (*control*), *wtap* knockdown (*wtap* RNAi), double knockdown of *wtap* and *grn* (*wtap + grn* RNAi), double knockdown of *wtap* and *cdk7* (*wtap + cdk7* RNAi) and double knockdown of *wtap* and *cdk9* (*wtap + cdk9* RNAi) planarians at 7 dpa. Dotted line, amputation plane (*n* ≥ 3). Scale bar, 200 μm. (H) Quantification of percentage of *pc2* positive regenerated neurons in each group of (G). Error bars represent standard deviation. Data are the mean ± SD (*n* ≥ 3 independent experiments). The *p* values were determined using a two‐sided unpaired Student's *t*‐test. See also Figures S5–S7.

To further investigate the functions of NP‐like cells in planarian regeneration, we analysed the effect of *wtap* knockdown on the expression of NP‐like cell markers. First, the IGV and enrichment analyses showed that the levels of m^6^A modification on *grn* and *ubiqp* transcripts were significantly decreased upon *wtap* knockdown at both 7 and 11 dpa (Figures 6A and S5C). Consistently, WTAP‐mediated m^6^A depletion leads to a drastic increase in *grn* and *ubiqp* expression in *wtap*‐deficient planarians (Figure S5B). Furthermore, through both whole‐mount fluorescent in situ hybridization and immunofluorescence assays, we observed an increase in *grn* mRNA and protein levels (Figure 6B–D), as well as increased colocalization of two markers of NP‐like cells, *grn* and *dph1* (Figure 6B,C). In addition, the expression of *dph1* has been restored by the double knockdown of *wtap* and *grn* (Figure S6A). It has been reported that GRN is a secretory protein important for neuronal functions^48^, ^49^, ^50^ and can exert function over a distance.^48^, ^51^ Furthermore, GRN can stimulate cell division by promote G1 phase to M phase directly.^52^ We found that the double knockdown of *wtap* and *grn* remarkably rescued the regeneration failure of planarians induced by *wtap* knockdown compared to the control (Figures 6B–F and S5E), even though *grn* knockdown did not affect regeneration (Figure S5F). Moreover, NP‐like cell cluster disappeared upon the double knockdown of *wtap* with *grn*, *cdk7* or *cdk9* as demonstrated by FISH staining of its markers (Figure 6B–D). In addition, co‐staining of BrdU and H3p revealed restored cell proliferation pattern and cell cycle state in the double knockdown worms of *wtap* with *grn*, *cdk7* or *cdk9* (Figure S6B–D). Notably, the whole mount FISH staining of *pc2* also showed restored regeneration of CNS in double knockdown worms (Figure 6G,H). Our single‐cell sequencing data showed that *Cyclin T1* (*ccnt1*), *cdk7* and *cdk9* have relative higher transcript abundance in the neoblast than any other cell types in the *wtap* knockdown planarians (Figure S7A), and NP‐like cells communicate with other cell types potentially through *grn* targeting on its receptor *sort1* based on cell–cell communication analysis using CellphoneDB (Figure S7B, C). These results suggest that GRN excreted by NP‐like cells might potentially act on neoblast. At the same time, for other cell–cell communication‐related gene transcripts containing m^6^A modification, such as *mtnr1b*, their ligands are also modified by m^6^A and upregulated upon *wtap* knockdown and seem to participate in planarian regeneration (Figure S7D, E). These data suggest that WTAP‐mediated m^6^A controls GRN expression levels, which further influences the regeneration of planarians.

## 3 DISCUSSION

Regeneration is a dynamic and tightly controlled process involving a complex yet well‐orchestrated gene regulation network. However, the potential significance of RNA m^6^A modification in regeneration is not well understood. Recently, one study reported that *kiaa1429* knockdown resulted in failed regeneration and decreased m^6^A level as well as accordingly changed gene expression pattern.^53^ In our study, we discovered that abundant RNA m^6^A modification exists in the planarian *Schmidtea mediterranea*, with a single high peak near the stop codon region and enrichment at CDS and 3′‐UTR region of mRNA, which are similar to the features in most model organisms. Interestingly, this m^6^A peak increases along the regeneration process, and functional analysis further demonstrated that the m^6^A‐modified genes are related to pathways of cell–cell communications, nervous system development, mRNA stability and cell cycle G2/M phase transition, suggesting important regulatory roles of m^6^A modification in planarian regeneration. Moreover, m^6^A depletion by *wtap* knockdown led to a complete loss of the regenerative ability of planarians attributing to the induction of cell cycle related genes *cdk7* and *cdk9* as well as the emergence of NP‐Like cell cluster with aberrant expression of cell–cell communication‐related secretory gene *grn*, identified by m^6^A sequencing analysis and rescue assays. Therefore, the signal axis of *wtap*‐m^6^A‐regulated *grn/cdk9/cdk7* determines the planarian regenerative potential through modulating cell–cell communication‐related gene expression.

RNA m^6^A modification has been reported to regulate stem cell differentiation and tissue regeneration.^27^, ^29^, ^36^, ^37^, ^38^, ^54^ It is required for axon regeneration by promoting protein translation of regeneration‐associated genes in the rodent model.^38^ Meanwhile, deletion of m^6^A reader YTH domain‐containing family protein 2 (YTHDF2) in mouse haematopoietic stem cells (HSC) was shown to facilitate HSC regeneration by enhancing the stability of mRNAs related to both Wnt signal and survival.^37^ In this study, using planarian *Schmidtea mediterranea* model system, we found the m^6^A peaks increase at CDS and 3′‐UTR junction during the regeneration progression. Moreover, knockdown of m^6^A regulatory unit, *wtap*, resulted in a significant reduction in mRNA m^6^A level and abolished the regenerative capability of planarian to regrow missing head and tail. These findings suggest that *wtap*‐mediated m^6^A is imperative for the whole‐organism regeneration of planarian.

Regeneration involves cell proliferation and the consequent generation of new tissues, which requires tight control of the cell cycle.^55^ In our study, we found that cell cycle‐related genes such as *cdk7*, *cdk9* and *ccnt1* are mainly expressed in neoblasts and upregulated upon *wtap* knockdown. Both CDK7 and CDK9 are members of the cyclin‐dependent protein kinase family with effector CDK activity to phosphorylate Pol II and other targets within the transcriptional machinery, and also serve as a CDK‐activating kinase for at least one other essential CDK involved in transcription.^56^, ^57^, ^58^ Both CDK7 and CDK9 have been shown to be required for the cell cycle regulation, such as the blockage of all cell divisions in *C. elegans* by *Cdk7* loss^43^ and G_1_ cell cycle arrest of *D.melanogaster* cells by RNAi‐mediated *Cdk9* silencing.^47^ Moreover granulin (grn)/progranulin has been shown to stimulate cell division,^52^ and *grn* inhibition decreases *gata1* expression and further the differentiation of erythroid in zebrafish.^59^ In support, we found an induced expression of *grn*, *cdk7* and *cdk9* post *wtap* knockdown accompanied by impaired regeneration, suggesting that m^6^A modification are critical for planarian regeneration through destabilizing the transcripts of these three genes.

Importantly, accumulative studies revealed that regeneration is a complicated process involving cell–cell communication in various tissue types.^60^ The flow of long‐range patterning information in regenerative morphogenesis is crucial to control stem cell behaviour in vivo.^61^ For example, muscle regeneration in response to injury, is a non‐specific inflammatory response to trauma, involving interaction between muscle and the immune system.^60^ Specifically, growth factors, such as insulin‐like growth factor I (IGF1), released by M1 macrophage, stimulate the growth of muscle progenitor cells at the terminal differentiation stage of regeneration. Notably, because of the limitation of GO terms annotated in planarian, we aligned the planarian transcriptome with the uniport database through blastx program, and selected the best comparison result. According to the comparison results and the related GO annotations, the GO annotations of planarian were enriched. And based on the GO analysis, we found that the cell communication pathway increased upon *wtap* knockdown. Finally, we identified a specific NP‐like cell cluster secreting growth factor GRN after *wtap* knockdown, suggesting that GRN might selectively target the neoblast to inhibit their differentiation into neural progenitor cells and block the eventual formation of individual organs.

Among all major cell types identified in planarian by scRNA‐seq,^17^, ^18^, ^20^ the most important cell type among them is the neoblasts, which can provide the cellular source for robust planarian regeneration. This totipotent stem cell can differentiate into all other cell types after injury or stimulation. Soon after amputation, neoblast starts to proliferate and migrate to wound site to form regenerative blastemal tissue, which becomes the foundation for re‐growing the missing tissue.^5^ However, both blastema formation and remodelling of pre‐existing tissue (morphallaxis) are required for the planarian regeneration.^55^ These intricate processes require correct cell–cell communications and precise coordination between stem cells and pre‐existing tissue to ensure the regeneration is finished on the right track.^5^ In our study, we identified a neural progenitor‐like cell cluster accumulated during the regeneration upon *wtap* knockdown, and further experiments demonstrated that the aberrant high expression of m^6^A modified gene, *grn*, secreted from this cell cluster disrupts the regeneration process. It is likely that such an abnormal cell cluster is supposed to be a group of neural progenitor cells at a special state during normal neural regeneration, which at the same time is also critical for subsequent events of regeneration of other tissue types to proceed. Therefore, we speculate that this special state of neural progenitor cell cluster, as we amplified its cell number by *wtap* knockdown, triggers a critical checkpoint regulated during the entire regeneration process that involves cell–cell communication mediated by GRN to ensure the correct path for regeneration, and *wtap* knockdown induced aberrant *grn* expression will stop the regeneration at this checkpoint.

As depicted in our model (Figure S7F), during planarian homeostasis, the expression level of *grn* is held at a moderate level to inhibit overgrowth, as equivalent to *grn* expression level before amputation at 0 hpa (Figure S5B). Upon injury, m^6^A modification selectively targets several important gene transcripts, including *grn* for degradation, manifested as its reduced expression level after 6 hpa (Figure S5B), as well as transcripts of cell cycle‐related genes including *ccnt1*, *cdk7* and *cdk9*. m^6^A‐mediated downregulation of these genes, especially the *grn*, may halt initial proliferation of stem cell pool at the first stage of regeneration, which in turn promotes their differentiation process, including the differentiation of neoblasts to the progenitor cells and eventually to different tissue types. After *wtap* knockdown, *grn* transcript accumulates to form a unique cell cluster resulting in increased GRN secretion, which may lead to cell cycle dysregulation and the loss of neoblast differentiation and the failure of planarian regeneration.

Nevertheless, the detailed mechanisms of cell–cell communication need further investigations and experimental proof. For example, it is uncertain how the expression levels of *grn*, *cdk7* or *cdk9* regulate the cell cycle and cell‐to‐cell communication, which eventually lead to regeneration failure. Also, it is worth noting that the *wtap* knockdown phenotype can produce not only regeneration‐specific phenotype, but also lysis phenotype under homeostatic conditions (data not shown). Therefore, it would be interesting to dissect further which *wtap*‐mediated m^6^A genes are regeneration specific. Other model systems which can harness overexpression systems may be able to answer these questions in depth.

Collectively, our study uncovered an essential role of WTAP‐mediated m^6^A modification in the regeneration of an entire organism. We further discovered that WTAP‐mediated m^6^A modification is particularly important for the neural progenitor cell population. So far, this process has not been well understood. Our study may have uncovered a novel mechanism in regulating planarian regeneration that utilizes GRN to monitor and permit the regeneration to happen correctly. Therefore, our study highlights the potential importance of cell–cell communication during the planarian regeneration and provides important insights for future studies in regenerative medicine.

## 4 MATERIALS AND METHODS

### 4.1 Planarian culture

Animals Clonal asexual (CIW4) and sexual strains of *Schmidtea mediterranea* were maintained in Montjuïch salts as previously described (1.6 mM NaCl, 1.0 mM CaCl_2_, 1.0 mM MgSO_4_, 0.1 mM MgCl_2_, 0.1 mM KCl and 1.2 mM NaHCO_3_ prepared in autoclaved Milli‐Q water).^62^ Animals were fed weekly with homogenized pig liver. All animals, 3–6 mm in length, were starved 1 week before any experiments.

### 4.2 Replication, size estimation and randomization

For every assay, at least three independent replicates with a minimum of three animals per experiment were performed. For RNAi phenotype characterization, numbers of animals used are indicated in each panel. No sample size estimation was performed. Animals for all experiments were randomly selected. All animals were included in statistical analyses, and no exclusions were done. Images were randomized before quantifications.

### 4.3 RNA interference

Double‐stranded RNA (dsRNA) was synthesized by in vitro transcription as previously described.^63^ In summary, whole worm cDNA was generated and subcloned into a vector. In vitro transcription was performed using T7 polymerase (Promega, P2075). Planarians were fed by dsRNA mixed with pig liver paste three times every 4 days and were amputated into three fragments pre‐ and post‐pharyngeal 24 h after the last feeding. For double knockdown of two genes (double RNAi), dsRNAs of two genes were mixed in liver paste each at the same final concentration as single gene knockdown assay, and delivered three times at a 4‐day interval. The *C*. *elegans unc‐22* gene was used as a negative RNAi control for single knockdown experiments, and was mixed with *wtap* dsRNA as control for double knockdown. The knockdown efficiency of double RNAi or single RNAi was verified by RT‐qPCR. Primers used for the dsRNA synthesis are listed in Table S6. ImageJ (V1.5) were used to measure the pixel area of regenerated blastema divided by that of the whole tissue fraction, and the same size of the amputated tissue fractions were chosen for quantification.

### 4.4 BrdU labelling and whole‐mount immunofluorescent staining

Animals were fed with 20 mg/mL BrdU (Sigma, 19160) as described before.^6^ Specimens were harvested after labelling for 6 h and fixed in 4% formaldehyde (FA). The rat anti‐BrdU (Abcam, ab6326) antibody was used for detection. Immunofluorescence staining was performed as described before.^63^ Different treatments were used for different antibodies. Following antibodies were used: Phospho‐histone H3 (Ser10; H3P; Abcam, ab32107). Muscle fibres were stained with the 6G10 antibody (1:1000, DSHB, 2C7; RRID:BDSC_9315). GRN protein was stained with anti‐GRN (1:1000, Proteintech, 10053‐1‐AP). Planarians were killed in 5% N‐acetyl cysteine (NAC) for 5 min at room temperature, and incubated in reduction buffer at 37°C for 8 min. After bleaching with 6% H_2_O_2_ overnight, planarians were washed with PBSTx (containing 0.3% Triton X‐100) over 6 h. The excretory system was stained with anti‐acetylated‐tubulin (1:1000, Sigma, T6793; RRID:AB_477585). Planarians were treated with 5% NAC for 5 min and 4% fixation for 20 min at room temperature followed by bleaching overnight and treated with proteinase K (1 μg/mL, Roche, 03115844001). After 2 h of blocking with 1% BSA, planarians were incubated with primary antibody overnight, then washed with PBSTx (containing 0.3% TritonX‐100) for more than 6 h. Blocking with 1% BSA for 1 h was performed before the fluorescent secondary antibody incubation overnight. After washing with PBSTx (containing 0.3% TritonX‐100) for more than 6 h, the slide was mounted with 80% glycerol containing Hoechst 33342 (10 μg/mL, Invitrogen, H3570). Quantification of fluorescent signals was analysed by ImageJ software (V1.5) as previously described.^64^

### 4.5 TUNEL assay

The TUNEL experiment was performed with ApopTag TUNEL Kit (Millipore, S7165) to determine apoptotic cell numbers in planarians following the manufacturer's instructions. Five per cent NAC (diluted in PBS) was used to remove the mucus coat of planarians. Planarians were fixed with 4% formaldehyde (with PBST containing 0.3% TritonX‐100) for 30 min at room temperature. The samples were then bleached in 6% H_2_O_2_ (diluted in PBST) in direct light overnight. The animals were incubated with TdT reaction mixture for 4 h at 37°C. The antibody was incubated for 4 h at room temperature. Animals were washed every half an hour for 4 h in PBST to reduce nonspecific labelling. Images were obtained with Leica SP8 confocal microscope. ImageJ was used for quantifications.

### 4.6 Whole‐mount in situ hybridization

DIG‐labelled riboprobes were synthesized using an in vitro transcription kit (Roche, 11277073910), according to the manufacturer's instructions. Animals were treated with 5% NAC for 5 min to clear epidermal mucus, and fixed in 4% formaldehyde in PBST for 30 min at room temperature. Then animals were dehydrated and stored in 100% methanol at −20°C for at least 1 h. The animals were bleached in 6% H_2_O_2_ under bright light overnight. Bleached samples were rehydrated through a graded series of methanol, and were incubated in probe mix at 56°C for 16 h. After the animals were washed and blocked, they were further incubated in anti‐Digoxigenin‐POD, Fab fragments (Roche, 11207733910; RRID:AB_514500) or anti‐Digoxigenin‐AP, Fab fragments (Roche, 11093274910; RRID:AB_514497) overnight at 4°C for 16 h, followed by extensive washing. For Colorimetric whole‐mount in situ hybridization, BCIP/NBT was used as substrates. For fluorescent development, a tyramide signal amplification system was used.^65^ For double‐colour FISH, POD inactivation was performed between signal developments in 100 mM NaN_3_ for 60 min. The primers used are listed in Table S6. Quantification of fluorescent signals was analysed by ImageJ software (V1.5) as previously described.^64^

### 4.7 Total RNA extraction

Total RNA was extracted from planarians with 1 mL TRIzol® reagent (Invitrogen, 15596018) through homogenizing on a tissue disruptor. mRNAs were purified from total RNAs using Dynabeads® mRNA Purification Kit (Ambion, 61006) and subjected to TURBO™ DNase (Invitrogen) treatment at 37°C for 30 min and ethanol precipitation. After centrifugation and extensive washing with 75% ethanol, the mRNA was dissolved and quantified using Qubit 3.0 (Thermo Fisher).

### 4.8 Western blot

Planarians were dissolved and homogenized in RIPA buffer (Cell Signalling Technology, 9806s) on a tissue disruptor. After incubation on ice for 10 min, the samples were centrifuged at 13,000 rpm for 30 min. Supernatant was collected and the Bradford method was used to measure protein concentrations. Western blot was performed as previously reported^66^ using the following antibodies: anti‐WTAP antibody (1:500, Proteintech, 10200‐1‐AP; RRID:AB_2216349), anti‐ß‐actin antibody (1:1000, Cell Signalling Technology, 4967s; RRID:AB_330288). The density of each band was quantified with ImageJ (version 1.8.0).

### 4.9 Quantitative reverse‐transcription PCR

To confirm knockdown efficiency and assess the relative RNA expression level, we conducted quantitative reverse‐transcription PCR (RT‐qPCR). All RNA templates were digested with TURBO™ DNase. cDNA synthesis was performed using the RevertAid™ First Strand cDNA Synthesis Kit (Invitrogen, 18064014). Takara SYBR Premix Ex Taq (Takara) was used according to the manufacturer's instructions and quantified by a CFX96 Real‐Time PCR System (Bio‐Rad). β‐actin was used as an internal control. Values of *p* were calculated using the two‐tailed unpaired Student's *t*‐test, ns, no significance. The primers used for qPCR are listed in Table S6.

### 4.10 UHPLC‐MRM‐MS/MS analysis

UHPLC‐MRM‐MS/MS analysis was performed as previously reported.^67^ In brief, 200 ng mRNAs were purified from planarian total RNA at indicated time points. The mRNAs samples were digested overnight with 0.1 U Nuclease P1 (Sigma‐Aldrich, N8630) and 1.0 U calf intestinal phosphatase (NEB, M0290) in a 0 μL reaction volume at 37°C. The mixture was subjected to UHPLC‐MRM‐MS/MS analysis to detect m^6^A after filtering the samples through ultra‐filtration tubes (MW cutoff: 3 kDa, Pall, Port Washington, New York). The Agilent 1290 UHPLC system coupled with the 6495 triple quadrupole mass spectrometer (Agilent Technologies) was used for the analysis. UHPLC separation of mono‐nucleosides was performed with a Zorbax Eclipse Plus C18 column (100 mm × 2.1 mm I.D., 1.8 μm particle size, Agilent Technologies). The mass spectrometer was operated in a positive ion mode. Three replicates were analysed for each sample, with an injection volume of 5 μL. The amount of m^6^A and adenosine (A) was determined by using a calibration curve as a standard. Nitrogen was used for nebulization and desolvation at 40 psi with a flow‐rate of 9 L/min, a source temperature of 300°C, capillary voltage of 3500 V and high purity nitrogen of 99.999%. The ribonucleoside standards for m^6^A and A were purchased from TCI, China.

### 4.11 RNA‐seq and MeRIP‐seq

mRNAs were purified from total RNAs using the Dynabeads® mRNA purification kit (Ambion, 61006). cDNA libraries were made according to the TruSeq RNA Sample Prep Kit (Illumina, FC‐122‐1001) protocol. All samples were sequenced by Illumina Nova‐seq with paired‐end 150 bp read length. For m^6^A MeRIP‐seq, the procedure is based on a published protocol.^68^ Briefly, purified mRNA was fragmented to a size of about 200 nt using the fragmentation reagent (Life Technologies, AM8740). Thirty microlitres of protein A magnetic beads (Thermo Fisher Scientific, 10002D) were washed twice in 1 mL IP buffer (150 mM NaCl, 10 mM Tris–HCl [pH 7.5], 0.1% NP‐40 in nuclease‐free H_2_O), resuspended in 500 μL IP buffer mixed with 5 μg anti‐m^6^A antibody (Millipore, ABE572) and incubated at 4°C with gentle rotation for at least 6 h. After two washes in IPP buffer (10 mM Tris–HCl [pH 7.4], 150 mM NaCl, 0.1% NP‐40 in DEPC‐treated), antibody‐bead mixture was resuspended in 500 μL of the IPP reaction mixture containing 500 ng fragmented mRNA, 100 μL of 5× IP buffer, and 5 μL of RNasin Plus RNase Inhibitor (Promega, N2611) and incubated for 2 h at 4°C. The beads were then washed twice each with 1 mL IPP buffer, low‐salt IPP buffer (50 mM NaCl, 10 mM Tris–HCl [pH 7.5], 0.1% NP‐40 in nuclease‐free H_2_O) and high‐salt buffer (500 mM NaCl, 10 mM Tris–HCl [pH 7.5], 0.1% NP‐40 in nuclease‐free H_2_O) for 10 min at 4°C. The beads were then eluted with 300 μL 0.5 mg/mL m^6^A in IPP buffer (with RNasin) with gentle rotation at RT for 1 h. The m^6^A‐modified RNAs were eluted using 200 μL of RLT buffer supplied in RNeasy Mini Kit (QIAGEN, 74106) for 2 min at room temperature. Supernatant was collected to a new tube while beads were pulled on magnetic rack. Four hundred microlitres of 100% ethanol was added to the supernatant. The mixture was applied to a RNeasy spin column and centrifuged at 13,000 rpm at 4°C for 30 s. The spin column was washed with 500 μL of RPE buffer, then 500 μL of 80% ethanol, and centrifuged at top speed for 5 min at 4°C to dry the column. m^6^A‐modified RNAs were eluted with 10 μL nuclease‐free H_2_O. For a second round of IP, eluted RNA was re‐incubated with new protein A magnetic beads prepared with new anti‐m^6^A antibody, followed by washes, elution and purification as above. Purified RNAs were used to construct library using the KAPA Standard RNA‐Seq Kit according to the manufacturer's instruction (KAPA, KR1139). Libraries were PCR amplified for 8–12 cycles and size‐selected on the 8% TBE gel. Sequencing was carried out on the Illumina Nova 6000 platform according to the manufacturer's instructions.

### 4.12 Single‐cell RNA‐seq library construction

For single‐cell RNA‐seq from 10× Chromium platform, Hoechst‐stained and PI‐negative cells (200,000 cells) from wild‐type animals, and cells from *wtap* knockdown animals were collected on ice using Influx sorter. Approximately 9000 counted cells were loaded per channel. The libraries were made using the Chromium platform and Chromium Single Cell 3′ v3 chemistry. Sequencing libraries were loaded on an Illumina nova 6000 flowcell with two 150 bp paired‐end kits.

### 4.13 RNA‐seq data analysis

Adaptor sequences were trimmed off for all raw reads using the Cutadapt software (version 1.2.1).^69^ Reads that were less than 18 nt in length or contained an ambiguous nucleotide were discarded by Trimmomatic (version 0.30).^70^ The remaining reads were aligned to the *Schmidtea mediterranea* transcriptome smed_20140614 using Bowtie2 (v2.2.9)^71^ default parameters. Counts were calculated for the sum of reads of each transcript. R package ‘DEseq2’^72^ was used to identify differentially expressed transcripts. Genes with different expression patterns (Figure 1B and Figure 4B) were defined by the K‐means algorithm in MEV software. In detail, genes were clustered by MEV with parameter—distance‐metric‐selection = Pearson correlation, number of cluster = N, maximum iterations = 50 and the gene expression level of each time point was compared to that of the previous one.^73^

### 4.14 MeRIP‐seq data analysis

For MeRIP‐seq, m^6^A‐enriched peaks in each m^6^A immunoprecipitation sample were identified by MACS2 peak‐calling software (version 2.0.10)^74^ with the corresponding input sample serving as control. MACS2 was run with default options except for ‘–nomodel’ to turn off fragment size estimation. A stringent cut‐off threshold for a *p*‐value of 0.001 was used to obtain high‐confidence peaks. In each stage, peaks with over 50% overlap in three biological replicates were used in the downstream analysis. The overlapping peaks were re‐annotated by the highest peak within the region of all three peaks. The m^6^A level with different patterns was defined by the K‐means algorithm in MEV software (version 4.4). In detail, genes were clustered by MEV with parameter–distance‐metric‐selection = Pearson correlation, maximum‐iterations = 50 and m^6^A level of each timepoint was compared to that of the previous one.^73^

### 4.15 Single‐cell RNA‐seq data processing

Reads were processed using the Cell Ranger 3.0.0 pipeline^75^ with default and recommended parameters. FASTQ files generated from Illumina sequencing were aligned to the *Schmidtea mediterranea* transcriptome smed_20140614 using the STAR algorithm.^76^ Next, Gene‐Barcode matrices were generated for each individual sample by counting unique molecular identifiers and filtering non‐cell associated barcodes. Only genes that can be translated into proteins were retained. We generated a gene‐barcode matrix containing barcoded cells and gene expression counts. This output was then imported into the Seurat (v3.1.2)^77^ R toolkit for quality control and downstream analysis of single‐cell RNA‐seq data. All functions were run with default parameters, unless specified otherwise. Low‐quality cells (<500 genes/cell, >6000 genes/cell, <15 cells/ gene and >20% mitochondrial genes) were excluded. In order to exclude multiple captures, which is a major concern in microdroplet‐based experiments, DoubletFinder (version 2.0.2)^78^ was employed to remove top N cells with the highest pANN score for each library separately, where N represents the doublet rates. Then all the datasets were merged using the ‘merge’ function in Seurat.

### 4.16 Identification of cell types and subtypes by nonlinear dimensional reduction

The Seurat package implemented in R was applied to identify major cell types.^77^ Highly variable genes were generated and used to perform PCA. Significant principle components were determined using JackStraw^77^ analysis and finally focusing on PCs 1–20. We grouped cell types based on their expression profile and matched them to known markers. To identify the subtype of neuronal cells, we used the reported markers of neuronal cells and also using ‘FindTransferAnchors’ and ‘TransferData’ function^77^ which provided by Seurat to predict the similarity between the subcluster and the reported cell types that identified by Plass.^18^ The gene name equivalencies beyond SMED to the transcriptome that used by Plass were transferred using the correspondence table which was downloaded from https://planosphere.stowers.org/chado/analysis?name=&program=&sourcename.

### 4.17 GO analysis

To enrich the GO annotation of planarian, we aligned the planarian transcriptome with the uniport database through blastx programme, and selected the best comparison result. According to the comparison results and the related GO annotation, the GO annotations of planarians were enriched. Then, GO analysis was performed using topGO,^79^ with all genes as background.

### 4.18 Cell–cell communication analysis

In order to explore cell–cell communication networks via ligand–receptor interactions, we initially identified the homologous genes of planarian and human genes by blastx (version 2.7.1).^80^ Then, we retained the genes with high homology with humans. For the gene corresponding to two annotations, we retained the result with the highest *e*‐value. After all, CellPhoneDB (v2.1.7)^81^ with default parameters (cellphonedb method statistical_analysis–threshold 0.01) was used to identify the interaction among cells and the related receptor–ligand pairs.

### 4.19 Quantification and statistical analysis

All statistical analyses of qPCR and imaging were performed at least three biological replicates. Student's two‐tailed unpaired *t*‐test was used for statistical comparisons and data were shown as mean ± SD. Values of *p* were used for significance analysis; ns, no significance. All statistical analyses were performed using GraphPad Prism (version 7.0).

## AUTHOR CONTRIBUTIONS

Yun‐Gui Yang conceived this project. Yun‐Gui Yang and Ying Yang supervised the study and analysed data. Guanshen Cui, Xin‐Yang Ge, Ge‐Ge Song, Xing Wang and Xiu‐Zhi Wang performed the experiments. Jia‐Yi Zhou and Bao‐Fa Sun performed bioinformatics analysis. Rui Zhang and Hai‐Lin Wang performed the UHPLC‐MRM‐MS/MS analysis. Yun‐Gui Yang, Ying Yang, Da‐Li Han, Wei‐Qi Zhang, Guanshen Cui, Jia‐Yi Zhou, Xin‐Yang Ge, Ge‐Ge Song, An Zeng, Yong‐Liang Zhao and Magdalena J. Koziol discussed and integrated the data. Qing Jing provided the planarians. Yun‐Gui Yang, Ying Yang, Guanshen Cui, Jia‐Yi Zhou, Xin‐Yang Ge, Ge‐Ge Song and Magdalena J. Koziol wrote the manuscript. All co‐authors provided feedback on the final manuscript.

## FUNDING INFORMATION

This work was supported by grants from the Strategic Priority Research Program of the Chinese Academy of Sciences, China (XDA16010501, XDA16010108), CAS Project for Young Scientists in Basic Research (YSBR‐073), the National Natural Science Foundation of China (91940304, 32121001, 31770872, 32070828, 31922017), the National Key R&D Program of China (2018YFA0801200), the Youth Innovation Promotion Association of CAS (Y2022040), the Beijing Nova Program (Z201100006820104, 20220484210), and Shanghai Municipal Science and Technology Major Project (2017SHZDZX01). This result is supported by the CAMS Innovation Fund for Medical Sciences (CIFMS) (2019‐I2M‐5‐015).

## CONFLICT OF INTEREST STATEMENT

The authors declare no competing interests.

## Supporting information


**Figure S1.** Changes of m^6^A modified genes during regeneration, related to Figure 2. (A) Histogram showing the percentage of mRNAs with different m^6^A peak numbers. The *x*‐axis represents the number of m^6^A peak on one mRNA and *y*‐axis represents the percentage. (B) Histogram showing the number of genes with m^6^A modification in different periods. (C) Venn plot showing the overlap of m^6^A‐modified mRNAs in five regeneration timepoints. (D) Venn plot showing the overlap of mRNAs from different clusters that shown in Figure 1B (yellow pool) and total m^6^A‐modified mRNAs at all timepoints (green pool). (E) Line chart showing one of the trends (first category) of mRNAs m^6^A level during regeneration, which with increased m^6^A level from 0 hpa to 7 dpa and decreased m^6^A level from 7 to 11 dpa. mRNAs with different expression pattern were defined by MEV with parameter––distance‐metric‐selection = Pearson‐correlation––number‐of‐cluster = 4––maximum‐iterations = 50. (F) Barplot showing the significant GO terms for genes shown in (E). (G) Line chart showing one of the trends (second category) of mRNAs m^6^A level during regeneration, which with increased m^6^A level from 0 hpa to 3 dpa and then decreased m^6^A level from 3 to 11 dpa. mRNAs with different expression pattern were defined by MEV with parameter––distance‐metric‐selection = Pearson‐correlation––number‐of‐cluster = 4––maximum‐iterations = 50. (H) Barplot showing the significant GO terms for genes shown in (G). (I) Line chart showing one of the trends (third category) of mRNAs m^6^A level during regeneration, with increased m^6^A level from 0 to 6 hpa and then decreased m^6^A level from 6 to 11 dpa. mRNAs with different expression pattern were defined by MEV with parameter––distance‐metric‐selection = Pearson‐correlation––number‐of‐cluster = 4––maximum‐iterations = 50. (J) Barplot showing the significant GO terms for genes shown in (I).
**Figure S2.** Detection of *wtap* knockdown efficiency and m^6^A methylation levels, related to Figure 3. (A) Western blot showing the knockdown efficiency of WTAP. Actin is used as a loading control. (B) Quantitative qPCR showing the relative expression of *wtap* in control and *wtap* knockdown planarians at 7 dpa. Error bars represent standard deviation. Data are the mean ± SD (*n* ≥ 3 independent experiments). The *p* values were determined using a two‐sided unpaired Student's t‐test. (C) UHPLC‐MRM‐MS/MS showing m^6^A level of mRNA extracted from control and WTAP knockdown (*wtap* RNAi) planarians at 7 dpa. Error bars represent standard deviation. Data are the mean ± SD (*n* ≥ 3 independent experiments). The *p* values were determined using a two‐sided unpaired Student's *t*‐test. (D) Bright‐field images showing WTAP knockdown (*wtap* RNAi) phenotype of head, trunk, tail tissue fragment at 1, 3, 5, 7 and 9 dpa. Scale bar, 500 μm. (E) Whole‐mount fluorescent in‐situ hybridization showing the expression and distribution of *pc2* and *smedwi‐1* transcript of trunk tissue fragment at 7 dpa. Arrow, amputated position. Scale bar, 500 μm. (F) Whole‐mount fluorescent BrdU immunostaining of control and *wtap* knockdown (*wtap* RNAi) planarians at 5 dpa. Dotted line, amputation plane. Scale bar, 300 μm. (G) Quantification of BrdU^+^ nuclei/mm^2^ at 7 dpa in (F). Error bars represent standard deviation. Data are the mean ± SD (*n* ≥ 3 independent experiments). The *p* values were determined using a two‐sided unpaired Student's t‐test. (H) Whole‐mount TUNEL assay showing the late apoptotic cells of control and *wtap* knockdown (*wtap* RNAi) planarians at 9 dpa. Scale bar, 200 μm. (I) Quantification of TUNEL^+^ nuclei/mm^2^ at 4 hpa, 1 dpa, 3 dpa, 5 dpa, 7 dpa and 9 dpa. Error bars represent standard deviation. Data are the mean ± SD (*n* ≥ 3 independent experiments). The *p* values were determined using a two‐sided unpaired Student's *t*‐test.
**Figure S3.** WTAP‐mediated m^6^A controls cell cycle‐ and cell–cell communication‐related factors, related to Figure 4. (A) Barplot showing the total number of up‐ and down‐regulated transcripts (*wtap* knockdown vs. *control* planarians) that with m^6^A modification at 0 hpa, 6 hpa, 3 dpa, 7 dpa and 11 dpa. (B) GSEA plots evaluating the changes in multicellular organismal signalling, compared *wtap* knockdown (*wtap* RNAi) with control samples at 3 dpa. Normalized *p* value = 0.05. (C) Whole‐mount fluorescent in situ hybridization showing the expression and distribution of *ccnb1* and *smedwi‐1* transcript of trunk tissue fragment at 5 dpa. Scale bar, 200 μm. (D) Barplot showing the enrichment of m^6^A modification on *cdk7* and *cdk9*. The *p* values were determined using Wilcoxon‐test. (E) Integrative genomics viewer (IGV) tracks displaying the distribution of MeRIP‐seq data that normalized by RNA‐seq data along *notum* mRNA in both control and *wtap* knockdown (*wtap* RNAi) samples. (F) IGV tracks displaying the distribution of MeRIP‐seq data that normalized by RNA‐seq data along *kcnd2* mRNA in both control and *wtap* knockdown (*wtap* RNAi) samples. (G) The phenotype of control (*control* RNAi), *cdk7* knockdown (*cdk7* RNAi) or *cdk9* knockdown (*cdk9* RNAi) planarians at 7 dpa. Bottom left number, planarians with phenotype of total tested. Scale bar, 500 μm.
**Figure S4.** Functional analysis in different cell types upon *wtap* knockdown during regeneration, related to Figure 5. (A) Violin plots showing expression levels of canonical markers for each cell types. (B) Barplot showing enriched gene ontology terms for up‐regulated genes upon *wtap* knockdown at 3 dpa in epidermal cells. (C) Barplot showing enriched gene ontology terms for up‐regulated genes upon *wtap* knockdown at 3 dpa in neoblast cells. (D) Heatmap showing enriched gene ontology terms for downregulated genes upon *wtap* knockdown in epidermal cells at different regeneration stages. The colour bar of heatmap represents *z*‐score of GO's *p* value. (E) Heatmap showing enriched gene ontology terms for downregulated genes upon *wtap* knockdown in neoblast cells at different regeneration stages. The colour bar of heatmap represents *z*‐score of GO's *p* value. (F) Heatmap showing enriched gene ontology terms for downregulated genes upon *wtap* knockdown in neuronal cells at different regeneration stages. The colour bar of heatmap represents *z*‐score of GO's *p* value.
**Figure S5.** Identification of NP‐like cell markers, related to Figure 6. (A) Expression level of *ubiqp* in control (*control*) and *wtap* knockdown (*wtap* RNAi) planarians during regeneration. (B) Lineplot showing the dynamic expression level of *grn* and *ubiqp* expression levels in both *control* and *wtap* knockdown planarians during the five regeneration stages. The expression level is quantified from bulk RNA‐seq. Error bars represent standard deviation. Data are the mean ± SD. The *p* values were determined using a two‐sided unpaired Student's *t*‐test. (C) Integrative genomics viewer (IGV) tracks displaying the distributions of MeRIP‐seq data that normalized by RNA‐seq data along *ubiqp* in both *control* (top) and *wtap* knockdown (bottom) planarians at 7 and 11 dpa. Barplot showing the enrichment of m^6^A modification on *ubiqp*. The *p* values were determined using Wilcoxon‐test. (D) IGV tracks displaying the distributions of MeRIP‐seq data that normalized by RNA‐seq data along *dph1* in both *control* (top) and *wtap* knockdown (bottom) planarians at 7 and 11 dpa. Barplot showing the enrichment of m^6^A modification on *ubiqp*. The *p* values were determined using Wilcoxon‐test. (E) qPCR showing the relative expression of *wtap* and *grn* in control (RNAi), *wtap* knockdown (RNAi) and double knockdown of *wtap* and *grn* at 7 dpa. Error bars represent standard deviation. Data are the mean ± SD (*n* ≥ 3 independent experiments). The *p* values were determined using a two‐sided unpaired Student's t‐test. (F) Phenotype of control and *grn* knockdown (*grn* RNAi) planarian at 7 dpa. Bottom left number, planarians with phenotype of total tested. Scale bar, 500 μm.
**Figure S6.** *wtap* regulates the regeneration of planarian through *cdk7*, *cdk9* and *grn*. Related to Figure 6. (A) qPCR showing the relative expression of *wtap*, *grn*, *cdk7*, *cdk9*, *dph1* and *ubiqp* in control (RNAi), *wtap* knockdown (RNAi), double knockdown of *wtap* and *grn* (*wtap + grn* RNAi) planarians at 7 dpa. Error bars represent standard deviation. Data are the mean ± S.D. (*n* ≥ 3 independent experiments). The *p* values were determined using a two‐sided unpaired Student's *t*‐test. (B) Whole‐mount fluorescent BrdU and H3p immunostaining of control, *wtap* knockdown (*wtap* RNAi), double knockdown of *wtap* and *grn* (*wtap + grn* RNAi), double knockdown of *wtap* and *cdk7* (*wtap + cdk7* RNAi) and double knockdown of *wtap* and *cdk9* (*wtap + cdk9* RNAi) planarians at 5 dpa. Dotted line, amputation plane. Scale bar, 300 μm. (C) Quantification of BrdU^+^ nuclei/mm^2^ of (B) at 5 dpa. Error bars represent standard deviation. Data are the mean ± S.D. (*n* ≥ 3 independent experiments). The *p* values were determined using a two‐sided unpaired Student's *t*‐test. (D) Quantification of H3p^+^ nuclei/mm^2^ of (B) at 7 dpa. Error bars represent standard deviation. Data are the mean ± SD (*n* ≥ 3 independent experiments). The *p* values were determined using a two‐sided unpaired Student's *t*‐test.
**Figure S7.** *wtap* regulates the regeneration of planarian by cell communication. Related to Figure 6. (A) Expression level of *ccnt1*, *cdk7* and *cdk9* in *wtap* knockdown (*wtap* RNAi) samples. (B) Network visualization showing neuronal cell communicates with other cell types through secreting GRN in *wtap* knockdown (*wtap* RNAi) samples. (C) Expression level of *sort1* in all of the single‐cell RNA datasets. (D) Network visualization of ligand‐receptor pair numbers among different cell types. The interaction among cells were calculated by CellphoneDB with default parameters (cellphonedb method statistical_analysis––threshold 0.01). (E) Network visualization of *mtnr1b* ligand‐receptor pair among different cell types in *wtap* knockdown (*wtap* RNAi) samples. (F) Working model for WTAP‐mediated m^6^A in regulating planarian regeneration.

**Table S1.** GO analysis of different expression genes.

**Table S2.** Genes with m^6^A modification at different time points.

**Table S3.** Cell classification in single‐cell data.

**Table S4.** During the regeneration, the cell proportions in control and wtap knockdown samples.

**Table S5.** The markers of NP‐like cells.

**Table S6.** Primers used for dsRNA synthesis, ISH and qPCR assays.

**Table S7.** GO analysis of different expression genes identified in single cell RNA sequencing data.

## Data Availability

The datasets including RNA‐seq, MeRIP‐seq, and single cell sequencing data generated and analyzed during the current study are available in the Genome Sequence Archive under accession number CRA004040 linked to the project PRJCA004712, and also the Gene Expression Omnibus database under accession number GSE171253.
