# Supplementary figures and images for: m6A promotes planarian regeneration
Source: Cell Prolif. 2023 Apr 21;56(5):e13481. doi: 10.1111/cpr.13481 (PMC10212710; doi:10.1111/cpr.13481)

Fig. S1

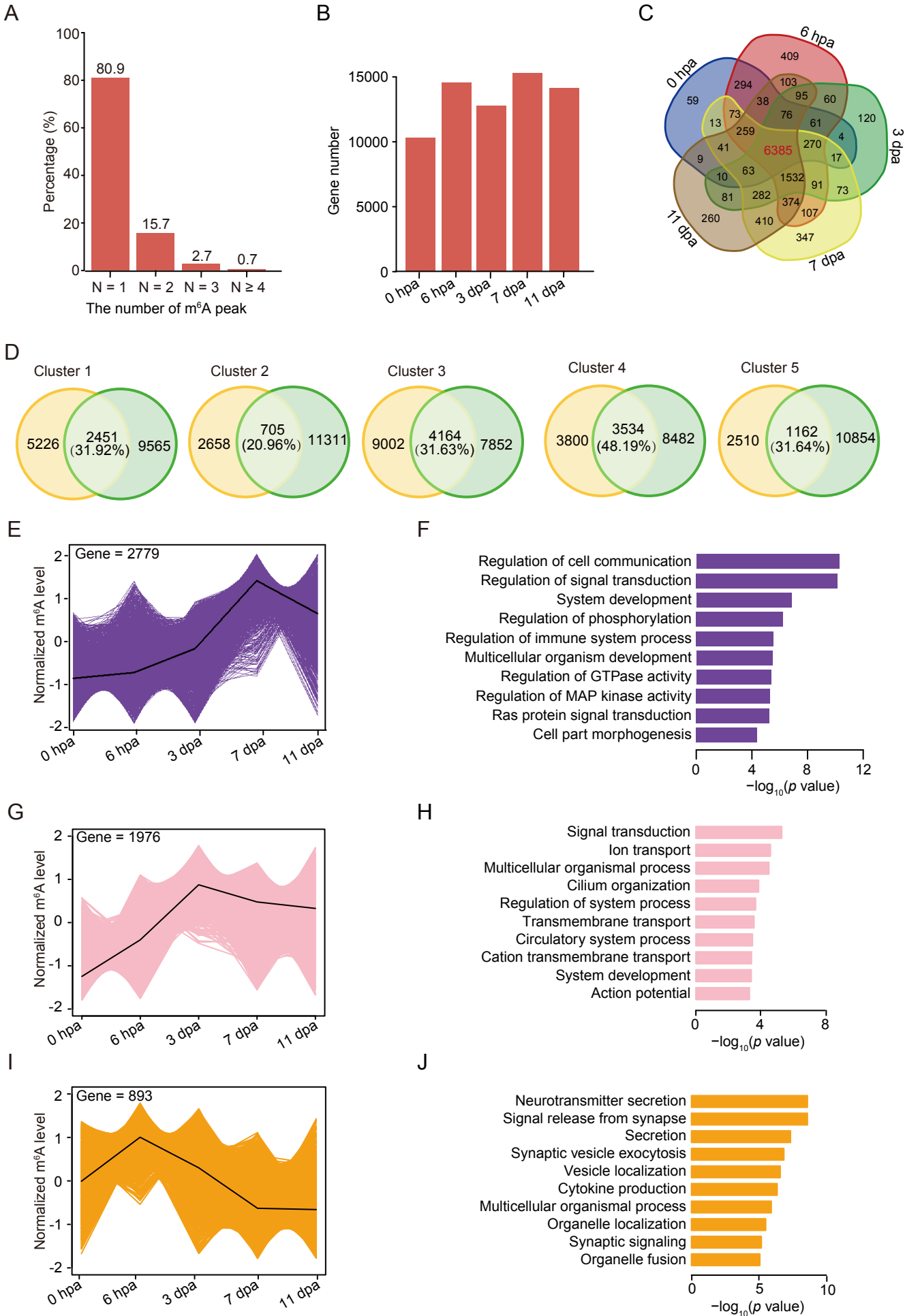

Fig. S2

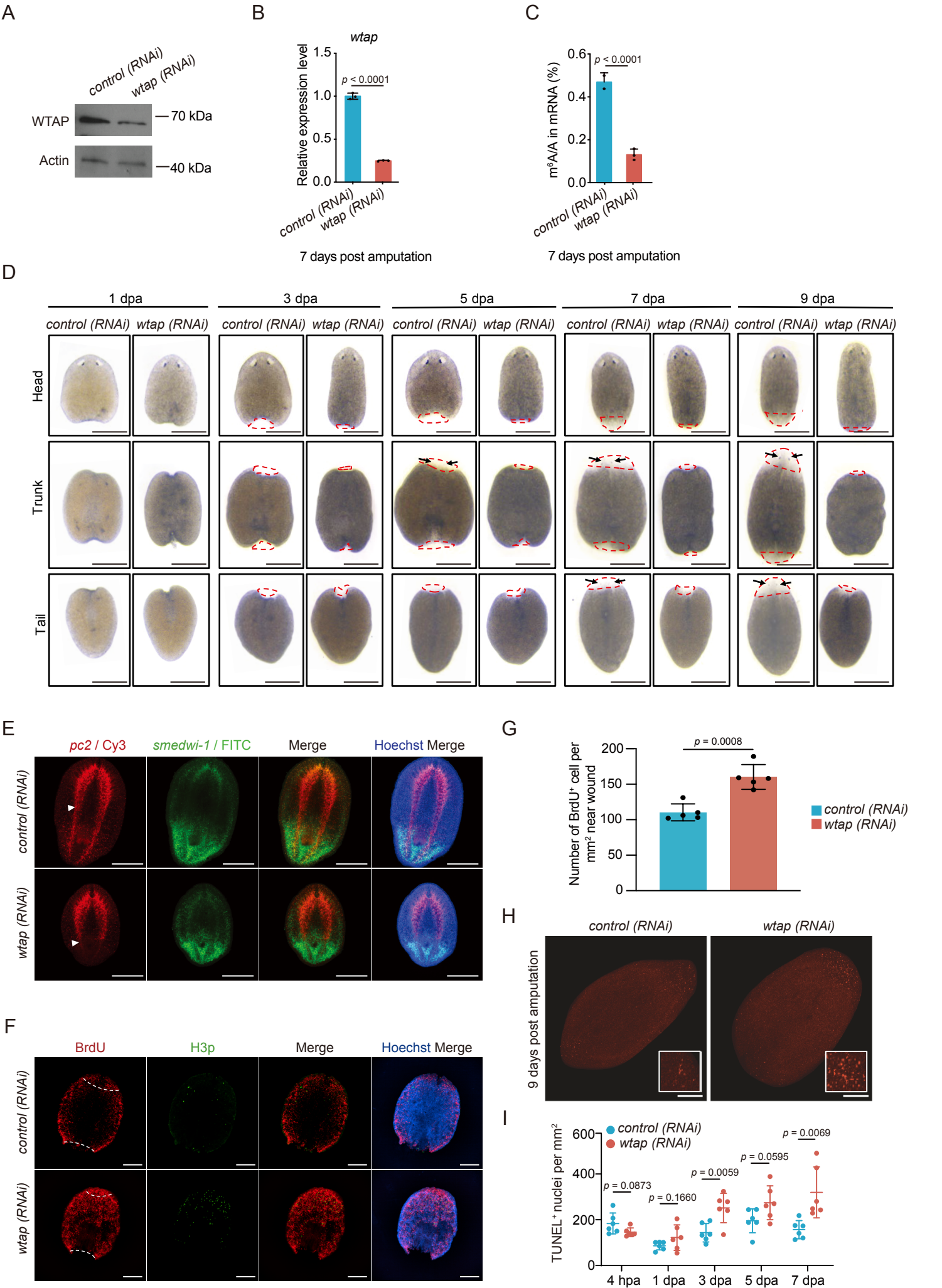

Fig. S3

A

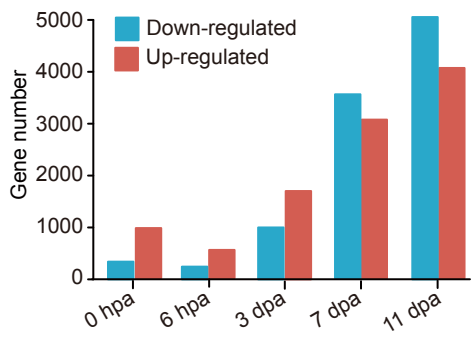

B

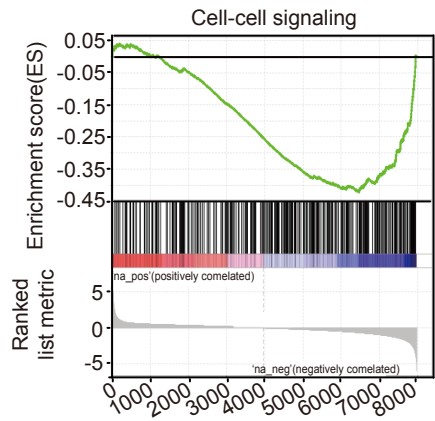

C

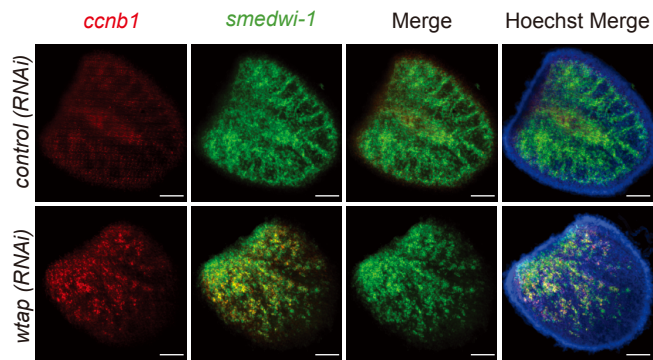

D

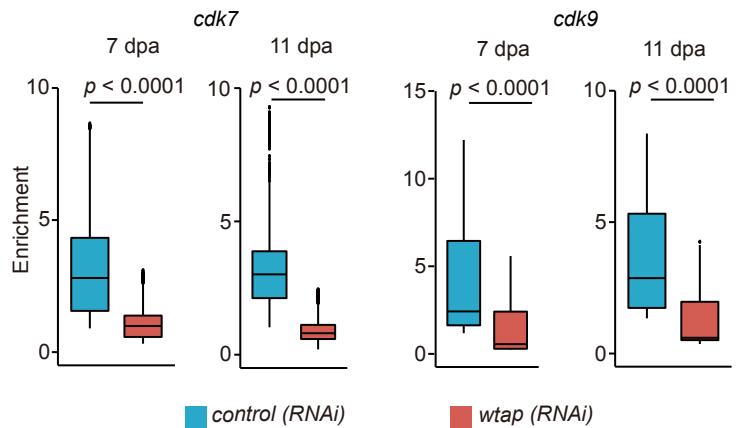

E

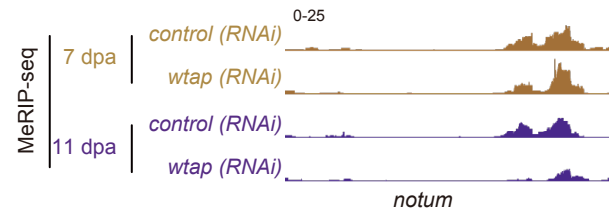

F

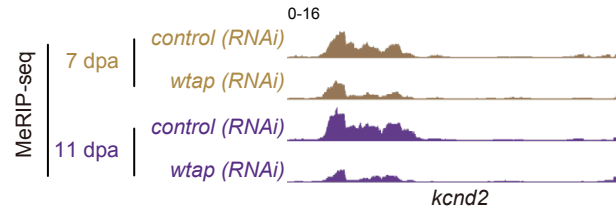

G

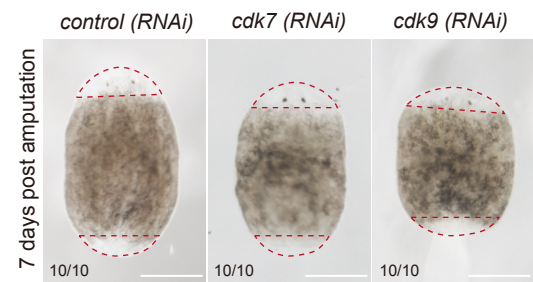

Fig. S4

A

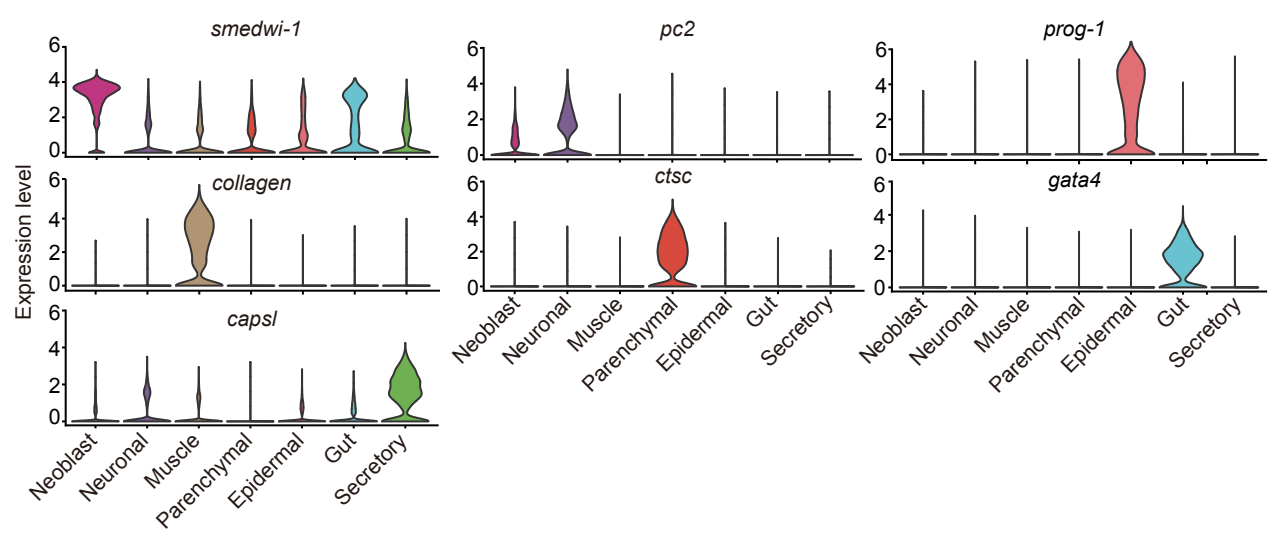

B

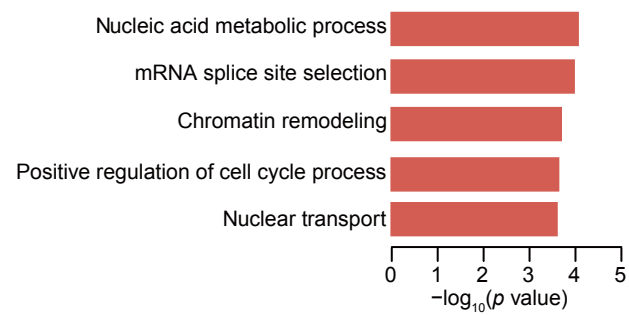

C

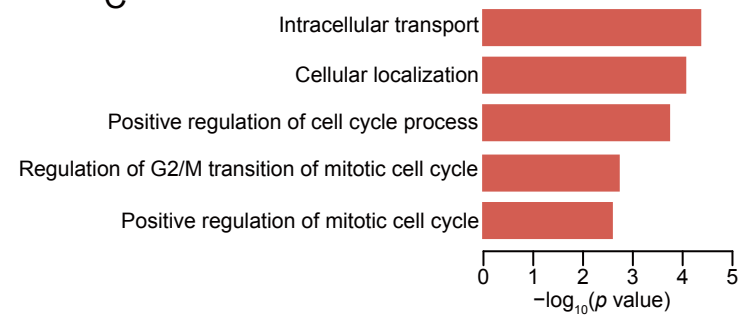

D

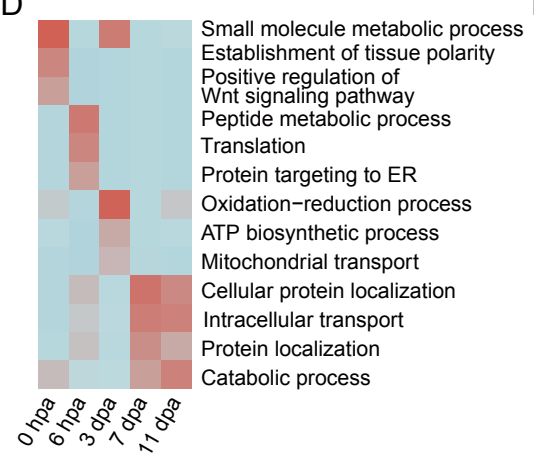

E

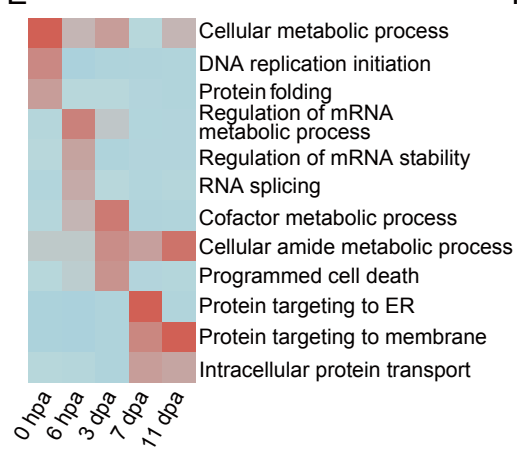

F

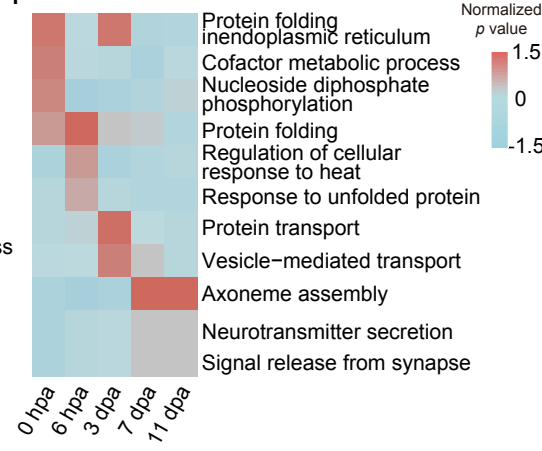

Fig. S5

A

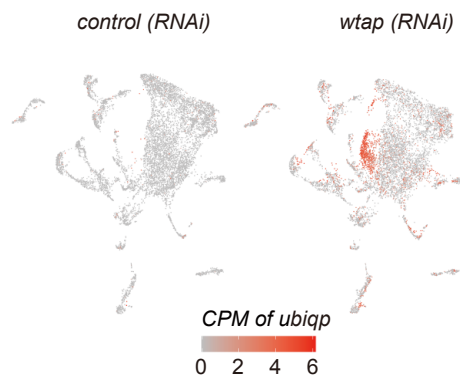

B

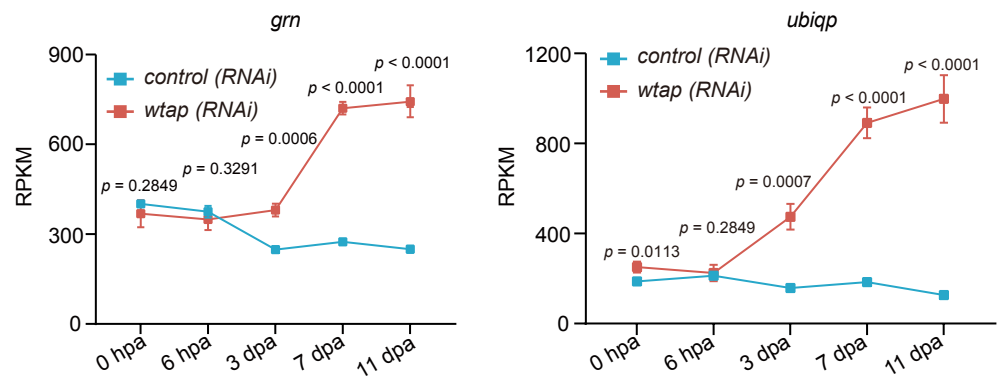

C

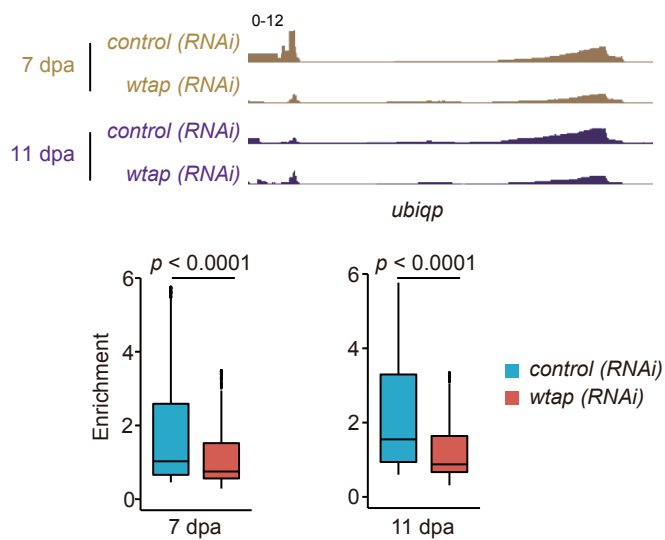

D

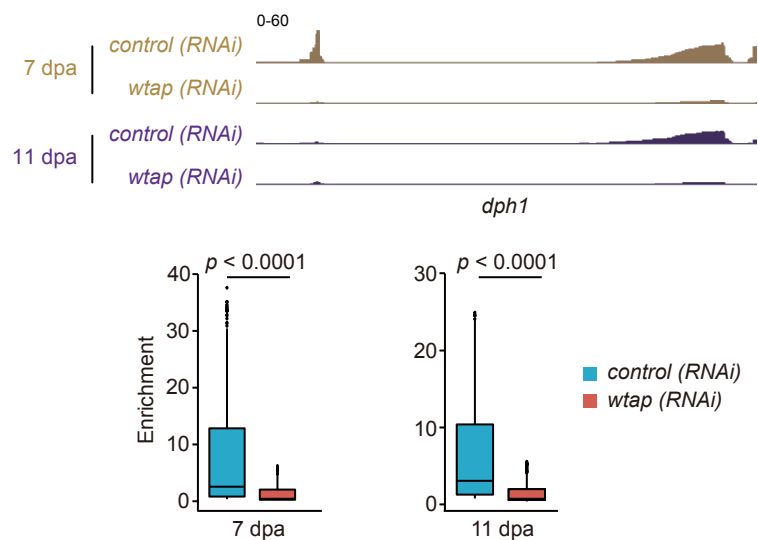

E

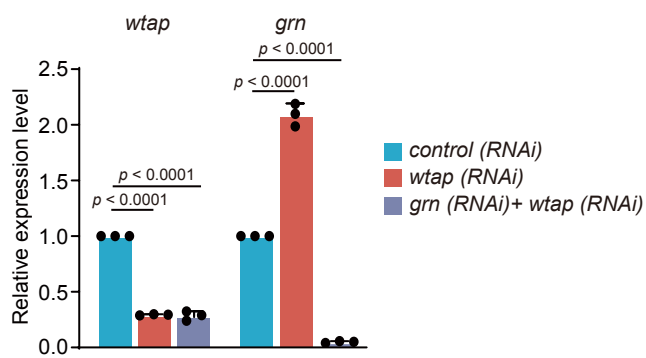

f

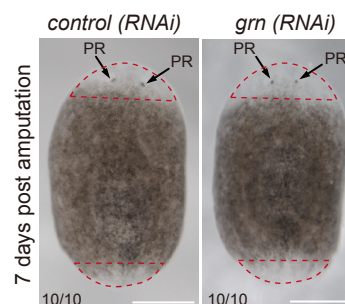

Fig. S6

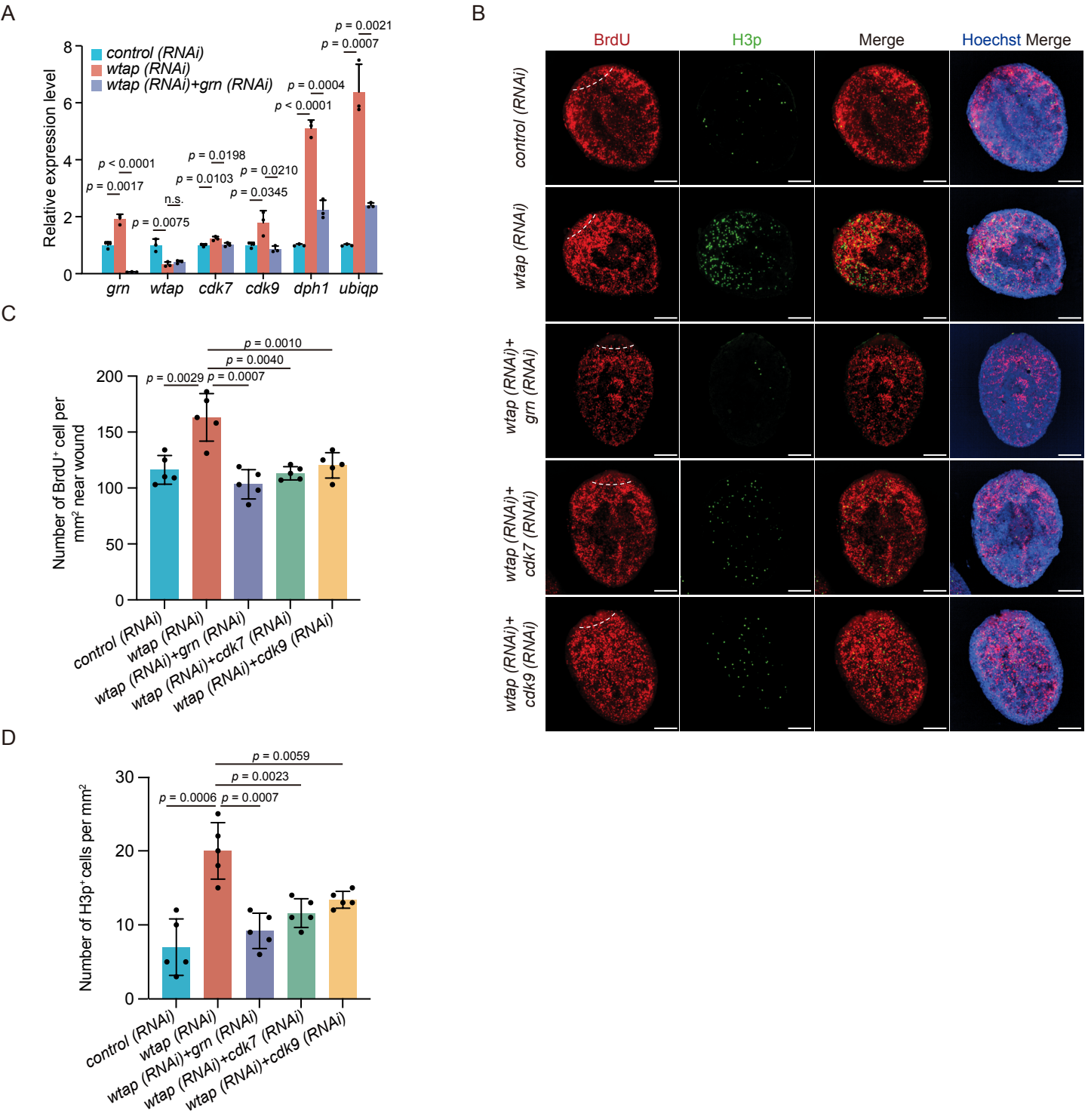

Fig. S7

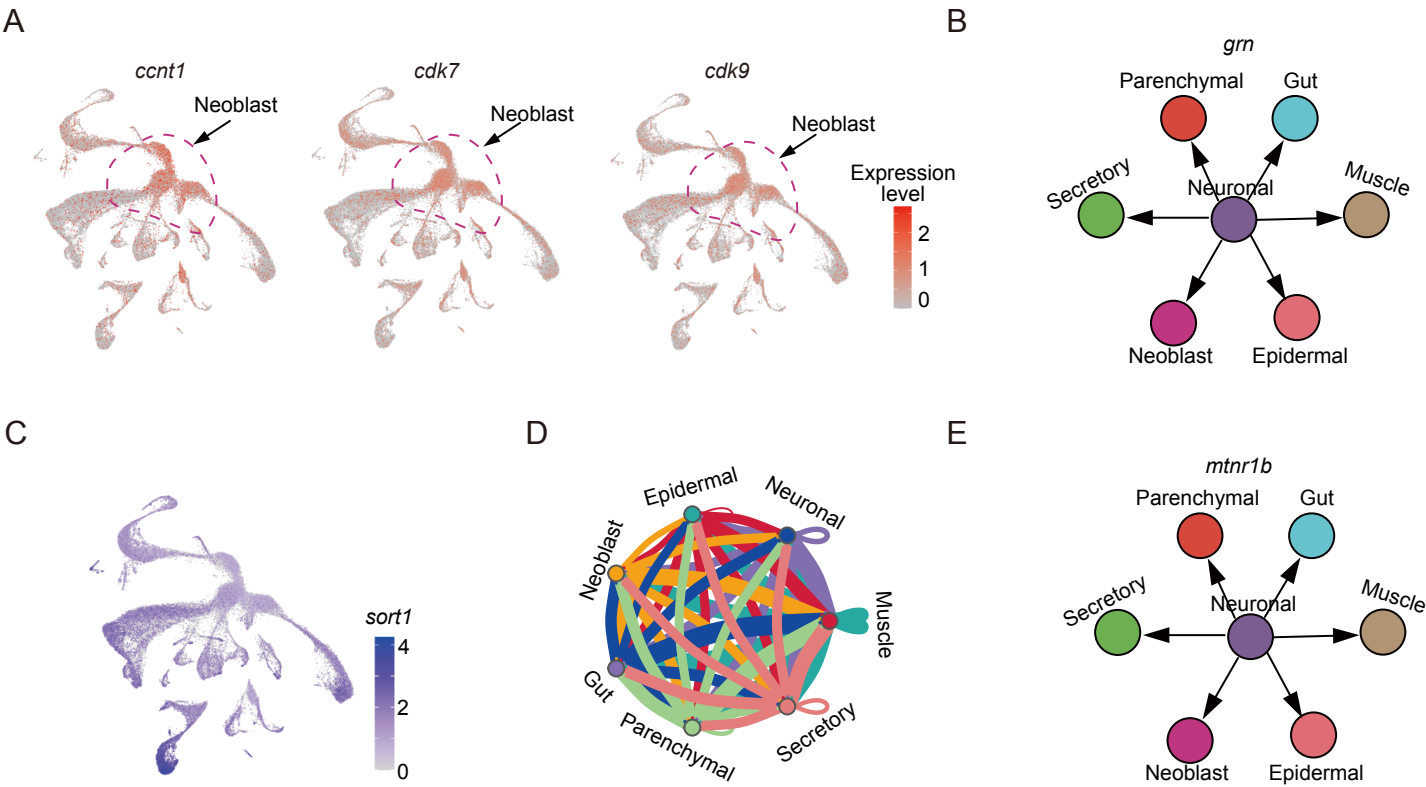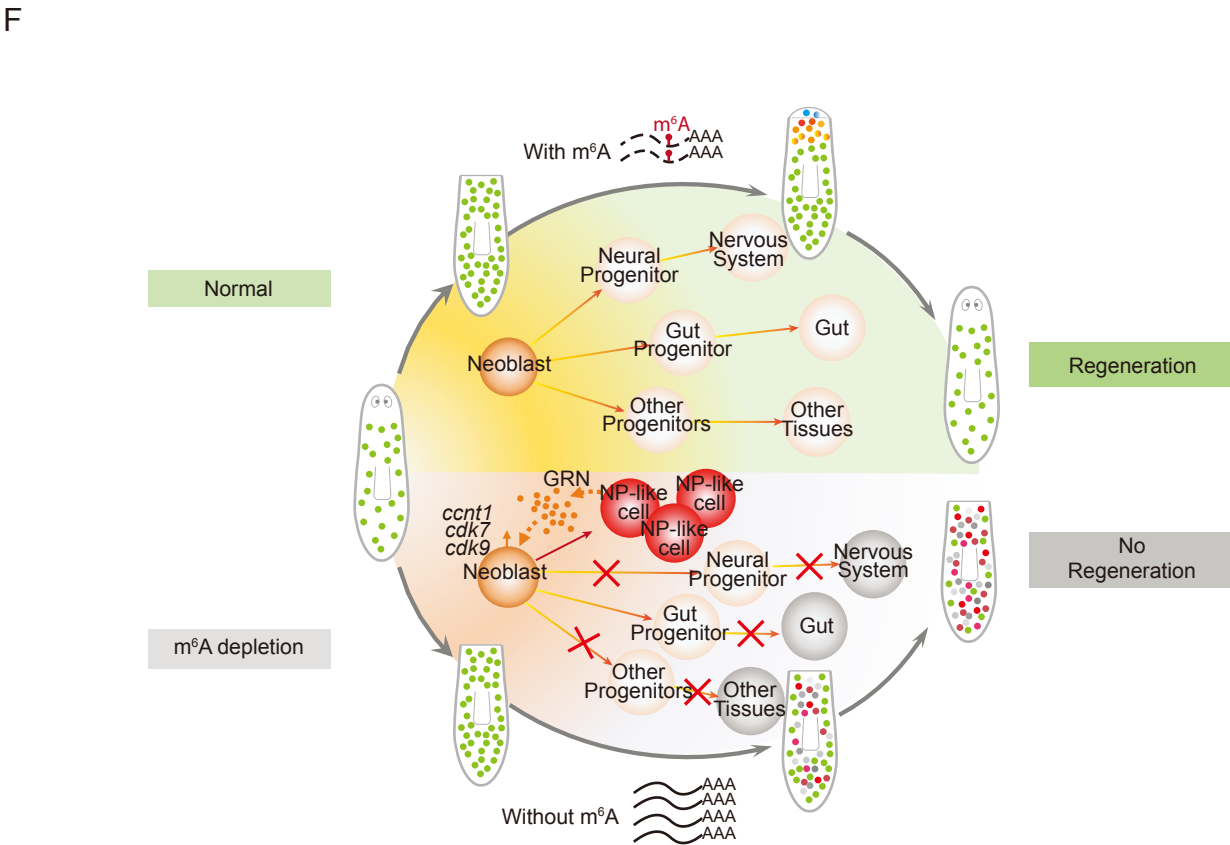

Supplement: Supplementary file 1 — Figure S1. Changes of m6A modified genes during regeneration, related to Figure 2. (A) Histogram showing the percentage of mRNAs with different m6A peak numbers. The x‐axis represents the number of m6A peak on one mRNA and y‐axis represents the percentage. (B) Histogram showing the number of genes with m6A modification in different periods. (C) Venn plot showing the overlap of m6A‐modified mRNAs in five regeneration timepoints. (D) Venn plot showing the overlap of mRNAs from different clusters that shown in Figure 1B (yellow pool) and total m6A‐modified mRNAs at all timepoints (green pool). (E) Line chart showing one of the trends (first category) of mRNAs m6A level during regeneration, which with increased m6A level from 0 hpa to 7 dpa and decreased m6A level from 7 to 11 dpa. mRNAs with different expression pattern were defined by MEV with parameter––distance‐metric‐selection = Pearson‐correlation––number‐of‐cluster = 4––maximum‐iterations = 50. (F) Barplot showing the significant GO terms for genes shown in (E). (G) Line chart showing one of the trends (second category) of mRNAs m6A level during regeneration, which with increased m6A level from 0 hpa to 3 dpa and then decreased m6A level from 3 to 11 dpa. mRNAs with different expression pattern were defined by MEV with parameter––distance‐metric‐selection = Pearson‐correlation––number‐of‐cluster = 4––maximum‐iterations = 50. (H) Barplot showing the significant GO terms for genes shown in (G). (I) Line chart showing one of the trends (third category) of mRNAs m6A level during regeneration, with increased m6A level from 0 to 6 hpa and then decreased m6A level from 6 to 11 dpa. mRNAs with different expression pattern were defined by MEV with parameter––distance‐metric‐selection = Pearson‐correlation––number‐of‐cluster = 4––maximum‐iterations = 50. (J) Barplot showing the significant GO terms for genes shown in (I). Figure S2. Detection of wtap knockdown efficiency and m6A methylation levels, related to Figure 3. (A) [file CPR-56-e13481-s005.pdf]
